# Bortezomib abrogates temozolomide-induced autophagic flux through an ATG5 dependent pathway

**DOI:** 10.3389/fcell.2022.1022191

**Published:** 2022-12-22

**Authors:** Mohummad Aminur Rahman, Agnete S. T. Engelsen, Shahin Sarowar, Christian Bindesbøll, Even Birkeland, Dorota Goplen, Maria L. Lotsberg, Stian Knappskog, Anne Simonsen, Martha Chekenya

**Affiliations:** ^1^ Department of Biomedicine, Faculty of Medicine, University of Bergen, Bergen, Norway; ^2^ Department of Oncology, Haukeland University Hospital, Bergen, Norway; ^3^ Department of Clinical Medicine and Centre for Cancer Biomarkers, Faculty of Medicine, University of Bergen, Bergen, Norway; ^4^ Department of Molecular Medicine, Institute of Basic Medical Sciences, Faculty of Medicine, University of Oslo, Oslo, Norway; ^5^ Department of Clinical Science, Faculty of Medicine, University of Bergen, Bergen, Norway; ^6^ Centre for Cancer Cell Reprogramming, Faculty of Medicine, University of Oslo, Oslo, Norway; ^7^ Department of Molecular Cell Biology, Institute for Cancer Research, Oslo University Hospital, Oslo, Norway

**Keywords:** glioblastoma (GBM), proteasome inhibitor bortezomib, secretome, temozolomide chemotherapy, cell cycle kinetics, autophagy flux, chemosensitization, apoptosis

## Abstract

**Introduction:** Glioblastoma (GBM) is invariably resistant to temozolomide (TMZ) chemotherapy. Inhibiting the proteasomal pathway is an emerging strategy to accumulate damaged proteins and inhibit their lysosomal degradation. We hypothesized that pre-treatment of glioblastoma with bortezomib (BTZ) might sensitize glioblastoma to temozolomide by abolishing autophagy survival signals to augment DNA damage and apoptosis.

**Methods:** P3 patient-derived glioblastoma cells, as well as the tumour cell lines U87, HF66, A172, and T98G were investigated for clonogenic survival after single or combined treatment with temozolomide and bortezomib *in vitro*. We investigated the requirement of functional autophagy machinery by utilizing pharmacological inhibitors or CRISPR-Cas9 knockout (KO) of autophagy-related genes -5 and -7 (ATG5 and ATG7) in glioblastoma cells and monitored changes in autophagic flux after temozolomide and/or bortezomib treatments. P3 wild-type and P3 *ATG5−/− (ATG5 KO)* cells were implanted orthotopically into NOD-SCID mice to assess the efficacy of bortezomib and temozolomide combination therapy with and without functional autophagy machinery.

**Results:** The chemo-resistant glioblastoma cells increased autophagic flux during temozolomide treatment as indicated by increased degradation of long-lived proteins, diminished expression of autophagy markers LC3A/B-II and p62 (SQSTM1), increased co-localisation of LC3A/B-II with STX17, augmented and no induction of apoptosis. In contrast, bortezomib treatment abrogated autophagic flux indicated by the accumulation of LC3A/B-II and p62 (SQSTM1) positive autophagosomes that did not fuse with lysosomes and thus reduced the degradation of long-lived proteins. Bortezomib synergistically enhanced temozolomide efficacy by attenuating cell proliferation, increased DNA double-strand breaks, and apoptosis in an autophagy-dependent manner. Abolishing autophagy in *ATG5* KOs reversed the bortezomib-induced toxicity, rescued glioblastoma cell death and reduced animal survival.

**Discussion:** We conclude that bortezomib abrogates temozolomide induced autophagy flux through an ATG5 dependent pathway.

## Introduction

Glioblastoma (GBM) is the most frequent, lethal primary brain malignancy in adults and is inherently chemoresistant. Standard therapy includes surgery, 75 mg/m^2^ temozolomide (TMZ) alkylating chemotherapy administered concomitantly with 2-Gy fractionated ionizing radiotherapy daily for 5 days in six consecutive cycles (chemoradiation with 60-Gy total radiation dose). Thereafter, patients continue with a 5-day treatment cycle of 150 mg/m^2^ TMZ for the first month, followed by 5 cycles of 200 mg/m^2^ for 5 days every month if well tolerated. Despite this aggressive treatment schedule, median survival is only 14.6 months (Stupp et al., 2005). Thus, there is an urgent, unmet need for strategies that can overcome chemoresistance as society ages, and GBM incidence might be expected to increase.

The cytotoxic effect of TMZ is mostly driven by the O^6^-methylguanine adducts that cause a preferential mismatch pairing of guanine with thymine rather than cytosine, leading to genomic instability. The generated single- and double-strand DNA breaks ultimately trigger cancer cell death by apoptosis (Agarwala and Kirkwood, 2000). However, a major mechanism that counteracts this cytotoxicity is the DNA repair enzyme, O^6^-methylguanine DNA methyltransferase (MGMT), which sequesters the toxic methyl adducts from O^6^ guanine prior to DNA replication (Pegg et al., 1995; Esteller et al., 2000), rendering the cancer cells resistant to TMZ cytotoxicity. Consequently, gene silencing by promoter methylation of the *MGMT* gene is a strong prognostic and predictive factor for response to TMZ (Hegi et al., 2005). However, *MGMT* promoter hypomethylation is not the only mechanism for chemoresistance, as GBMs also harbour mutations in base excision repair genes (Johannessen et al., 2013; Agnihotri et al., 2014) that may underlie the heterogeneity in patients’ responses to TMZ. This may explain why some tumors are resistant to TMZ despite the methylated *MGMT* promoter. Moreover, recurrent tumors may acquire a chemo-resistant phenotype denoted by whole genome enrichment of the C:G>T mutational signatures. A further gain of inactivating mutations in components of the mismatch repair pathway (Wang et al., 2016) is also indicative of TMZ-induced mutagenesis.

Notwithstanding genetic alterations that promote drug resistance, a common survival strategy for many cancers is metabolic reprogramming characterized by increased macroautophagy (Gopal et al., 2014; Perera et al., 2015; Klionsky et al., 2021) (hitherto referred to as autophagy), a well-conserved catabolic process that allows sequestration of long-lived cellular proteins, toxic protein aggregates, and damaged organelles into double-membrane autophagosomes. The cargo is subsequently degraded after the fusion of the autophagosomes with lysosomes to form autolysosomes (Seglen et al., 1980; Galluzzi et al., 2017; Negi et al., 2022). This process will henceforth be referred to as autophagic flux. The formation of autophagosomes is a multistep process involving several proteins, including the serine/threonine Unc-51-like Autophagy-Activating-Kinases 1 and 2 (ULK1 and ULK2), forming a multi-subunit ULK-complex, which initiates the formation of the phagophore membrane (Mizushima et al., 2010; Kim et al., 2011; Tsuboyama et al., 2016) by recruiting the Vacuolar Protein Sorting-associated protein (VPS34, a class III phosphatidyl ionositol-3 kinase) and downstream proteins that further promote elongation and closure of the phagophore to form an autophagosome (Mizushima et al., 2011). Protein products of several autophagy-related genes (ATG), including ATG5 and ATG7, mediate conjugation of the ubiquitin-like microtubule-associated protein 1A/1B-light chain 3 (MAP1LC3A/B, hereafter referred to as LC3), to phosphatidylethanolamine in autophagic membranes (referred to as LC3A/B-II). LC3A/B-II can interact with the autophagy receptor p62/Sequestosome-1 (p62/SQSTM1), which further recruits ubiquitin-tagged cargo into autophagosomes. Fusion of the latter with acid hydrolase containing lysosomes is controlled by SNARE proteins, including syntaxin 17 (STX17), resulting in the formation of autolysosomes where cargo becomes degraded into components that can be reused by the cell (Mizushima et al., 2010).

Autophagic flux is thus postulated to function as an adaptive survival mechanism that sustains cancer cells during conditions of stress (Simonsen et al., 2008; Fitzwalter and Thorburn, 2015; Galluzzi et al., 2015; Pietrocola et al., 2016). Transcriptional regulation by members of the forkhead homeobox-type O (FOXO), as well as deregulated signaling pathways involving the MEK/ERK and PI3K are known to affect basal rates and dependence on autophagic flux in different conditions and cancer types (Kim et al., 2011; Perera et al., 2015; Fitzwalter et al., 2018; Tompkins and Thorburn, 2019). Indeed, increased autophagic flux after TMZ treatment has been proposed to represent an additional mechanism for TMZ resistance (Pawlowska et al., 2018) in GBM cells (Kanzawa et al., 2004; Natsumeda et al., 2011; Harder et al., 2019; Zhu et al., 2019). As a result, the combination of anti-cancer treatments with FDA-approved inhibitors of autophagy flux, such as chloroquine or hydroxychloroquine, has been tested in clinical trials (Mulcahy Levy and Thorburn, 2020) to potentiate the therapeutic efficacy of TMZ (Kanzawa et al., 2004; Golden et al., 2014; Tompkins and Thorburn, 2019).

Cancer cells may nevertheless acquire resistance to death by abrogated autophagy, serendipitously rendering them more susceptible to proteasome inhibition (Towers et al., 2019). Inhibiting the 26S proteasome has emerged as an attractive strategy to accumulate damaged or misfolded proteins, reactive oxygen species, and endoplasmic reticulum stress, ultimately lowering the apoptosis threshold of cancer cells (Adams et al., 1999; Goldberg, 2003; Pawlowska et al., 2018). New generation reversible and non-reversible proteasome inhibitors, such as Ixazomib and Marizomib, respectively, are currently under clinical investigation in combination with anti-neoplastic treatments in cancers, including GBM. Like Ixazomib, Bortezomib (BTZ, Velcade) reversibly blocks the chymotryptic-like activity of the β1 and β5 subunits of the 26S proteasome. BTZ is approved for the treatment of multiple myeloma and mantle cell lymphoma (Stapnes et al., 2007) and has been explored in early phase clinical studies for GBM (Kubicek et al., 2009; Friday et al., 2012; Bota et al., 2013; Kong et al., 2018; Rahman et al., 2020). *In vitro* studies with BTZ alone or in combination with other drugs showed potent anti-cancer activity against various malignancies through multiple mechanisms (Frankel et al., 2000; Nawrocki et al., 2002). We demonstrate here that pre-treatment with BTZ prior to TMZ potently sensitized GBM cells to TMZ, as indicated by reduced IC_50_ doses, rapidly abrogated autophagic flux and strongly induced S-phase DNA damage and apoptosis in an ATG5 dependent manner.

## Material and methods

### Isolation of primary glioma cells

P3 patient derived GBM cells were expanded from biopsies obtained from patients undergoing tumour resection at the neurosurgery department (Haukeland University Hospital; Bergen, Norway), following biobank (REK Vest 013.09/20879) and project (REK 2018/71) approval from the regional ethical committee, the Norwegian Data Protection Agency and patient informed consent. These cells, as well as long-term established U87, A172, and T98G cell lines (ATCC, Manassas, VA, United States) and HF66 (Henry Ford Institute, Detroit, MI, United States) were propagated in Dulbecco’s modified eagle medium (DMEM, Sigma-Aldrich; St. Louis, MO, United States) supplemented with 10% fetal bovine serum, non-essential amino acids, 100 U/mL penicillin/streptomycin and 400 μM l-glutamine (complete medium; all Cambrex; East Rutherford, NJ, United States) at 37°C in a humidified atmosphere of 5% CO_2_.

### Drugs

Temozolomide (2706/50, Tocris Bioscience, Bristol, United Kingdom) was dissolved in dimethyl sulfoxide (DMSO and stored at –20°C. Bortezomib (Velcade^®^, 576415, Janssen, Norway) (Haukeland University Hospital pharmacy, Bergen, Norway) was dissolved in 0.9% v/v sodium chloride and stored at –80°C. ULK1 inhibitor (MRT68921, Cat. No. S7949, Selleckchem, United States) and VPS34 inhibitor (VPS34-IN1, Cat. No. S7980, Selleckchem, United States) were dissolved in DMSO and stored at –20°C. Bafilomycin A1 (Cat. No. SML1661) and Chloroquine diphosphate salt (Cat. No.C6628) were obtained from Sigma-Aldrich. Chloroquine was dissolved in water and stored at –20°C.

### Clonogenic survival assay

Cells were seeded at plating efficiency density (Franken et al., 2006) (1000 cells/well) in 6-well plates and exposed to temozolomide (72 h: 6–500 µM) or BTZ (48 h: 1–25 nM) or 1 h incubation with MRT68921 (1 µM) or VPS34-IN1 (1 µM), monotherapy or in combination, followed by further observation for 14 days. Colonies were stained with crystal violet and counted as previously described (Franken et al., 2006; Svendsen et al., 2011). IC_50_ was calculated using Prism software version 6.07 (GraphPad; La Jolla, CA, United States). Compusyn software was used for drug combination synergy calculation following the Chou-Talalay method(Chou and Talalay, 1984). All experiments were performed in triplicate and repeated at least 3 independent times.

### Western blot analysis

Cells were lysed in Kinexus lysis buffer: 20 mM MOPS, 5 mM EDTA, 2 mM EGTA, 30 mM NaF, 0.5% Triton X, 1 mM PMSF, pH 7.2, protease inhibitor (cocktail tablet, Roche; Basel, Switzerland), and phosphatase inhibitor (cocktail tablet, Roche). Samples (10–20 µg) were run on SDS/PAGE with NuPAGE precast 4–12% gradient gels (Invitrogen; Carlsbad, CA, United States) and blots were incubated overnight at 4°C with primary antibodies (Supplementary Table S1) followed by incubation for 1.5 h at room temperature (RT) with a species specific secondary HRP-conjugated antibody diluted 1:10000. Chemiluminescence detection was performed with Super Signal West Femto Maximum Sensitivity Substrate (Thermo Fisher Scientific, Bremen, Germany) on the LAS-3000 (Fujifilm Medical Systems Inc.; Stamford, Connecticut, United States). Relative protein expression levels were normalized to GAPDH and quantified using ImageJ software (NIH; Bethesda, MD, United States).

### Cell cycle distribution and kinetics

P3 and T98G cells with and without CRISPR-Cas9 ATG5−/− and ATG7shRNA knockdown were treated with TMZ (72 h: 50 μM and 100 µM for P3; 125 μM and 250 µM for T98G) or BTZ (48 h: 10 nM) monotherapy or in combination. Briefly, for cell cycle distribution analysis, cells were harvested post-treatment, fixed in ice-cold ethanol, and stained with 50 μg/ml propidium iodide (PI) or phospho-histone H3 (Ser10) (Supplementary Table S1), as previously described (Svendsen et al., 2011). Data were acquired on a FACS Accuri™ flow cytometer (BDbiosciences; Franklin Lakes, NJ, United States), and analyzed with FlowJo^®^ software (Treestar Inc.; Ashland, OR, United States). The Dean-Jett-Fox algorithm with synchronized peaks was used to analyze the DNA histograms of single cells from three independent experiments.

### Evaluation of treatment-induced DNA double-strand breaks (DSBs) by flow cytometry

The BD Pharmingen TM BrdU Flow kit (FITC BrdU Flow kit, cat.no 557891, BD Pharmingen, San Jose, CA, United States) was applied to evaluate treatment-induced DNA double-strand breaks (DSBs) by flow cytometry according to the manufacturer’s instructions. Briefly, cells were cultured and treated as described prior to a 90-min pulse by 10 µM bromodeoxyuridine (BrdU, BD Pharmingen) to enable the detection of cells actively synthesizing DNA. The cells were fixed and propagated for staining with FITC-labelled anti-BrdU antibody and APC-labelled pH2AX (Ser139) antibody (cat.no 12-4207, clone 1B3, Abeomics, San Diego, United States), according to the manufacturer’s protocol (BD Pharmingen). 7-AAD counterstain (BD Pharmingen) was used to visualize DNA content. Cells were gated based on FSC-A SSC-A dot plots, and single cells on FSC-A, FSC-H dot plots as previously described (Svendsen et al., 2011). The S-phase cell population was gated from the DNA (7-AAD) and BrdU (FITC) plots to evaluate the pH2AX (APC) expression within the population of S-phase cells. Mean values of 3 replicates + -SD are shown for each experimental condition.

### Autophagic flux assay

The autophagic flux assay was carried out using the Premo™ Autophagy Tandem Sensor RFP-GFP-LC3 Kit (P36239, Molecular Probes, Thermo Fisher Scientific, Bremen, Germany), following the manufacturer’s instructions. Briefly, 2.5×10^4^ P3 cells were cultured on coverslips in 24-well plates overnight, incubated with 10 μl of BacMam reagent for 24 h, and then treated according to schedule with BTZ (10 nM), TMZ (50 and 100 µM) or in combination. 50 μM Chloroquine (CQ) was added 24 h before end of the BTZ, TMZ or combination treatments. Cells were washed, slides mounted with Prolong Gold DAPI (P36931, Molecular Probes) and confocal images acquired on a Leica T SC SP5 3X (Leica, Wetzlar, Germany). The number of LC3B-positive autophagosomes and autolysosomes in merged images was quantified in at least 10 cells from each treatment group.

### Long-lived protein degradation

P3 and T98G cells were seeded at a density of 4 × 10^4^ cells/well in 24-well plates and treated with BTZ, TMZ, or in combination. To measure the degradation of long-lived proteins by autophagy, proteins were first labeled for 48 h with 0.25 μM Ci ml^−1^ L-^[U−14C]^ VALINE (Perkin Elmer, Waltham, MA, United States) in GIBCO-RPMI 1640 medium (Invitrogen, Carlsbad, CA, United States) containing 10% FBS, washed in warm PBS and then chased for 18 h without radioactivity in DMEM containing 10% FBS and 10 mM valine (Sigma-Aldrich), to allow degradation of short-lived proteins as previously described (Holland et al., 2016). 50 μM CQ was added 24 h before cell harvest. After 24 h, the supernatant was collected, 50% TCA was added, and proteins precipitated over-night at 4°C. The cells were lysed with 0.2 M KOH over-night at 4°C. The supernatant was centrifuged and transferred to a new tube, the precipitate dissolved and moved to the same sample as the cell lysate. Both supernatant and lysate were moved to separate counting vials and mixed with 3 ml scintillation fluid (Ultima Gold #6013321, Perkin Elmer). ^14^C levels were measured in each sample using a Packard Liquid Scintillation Analyzer. The percent degradation was calculated by comparing the amount of ^14^C in the supernatant to the total ^14^C levels (supernatant and lysate). The autophagic flux was calculated by subtracting the percent degradation of the CQ treated sample from the untreated sample, for each culture medium. All samples were related to the control.

### Generation of ATG5 knockout cells using CRISPR-Cas9 genome editing

Human *ATG5* knockout P3 and T98G GBM cell lines were generated by using LentiCRISPRv2 (Imam et al., 2016) (purchased from Addgene, plasmid# 99573). Plasmids were transfected into 293T cells using BBS/CaCl_2_ to produce lentivirus. The viral supernatant was harvested at 48 h post-transfection, filtered through 0.45-μm filters (Millipore). Infection of P3 and T98G cells was performed by centrifugation of cells with virus at 2225 rpm for 90 min at room temperature in the presence of 10 μg/ml polybrene (Sigma-Aldrich). Enrichment selection using puromycin (1 μg/ml) (Sigma-Aldrich) was done and collected for gene knockout assessment by western blotting.

### ATG7 RNA interference

Human ATG7 shRNA-GFP (5′- CAG​TGG​ATC​TAA​ATC​TCA​AAC​TGA​T-3′) and scrambled control shRNA-GFP (5′- GGG​TGA​ACT​CAC​GTC​AGA​A -3′) lentiviral plasmids were purchased from Applied Biological Materials Inc. (Richmond, BC, Canada). Plasmids were transfected into 293T cells using BBS/CaCl_2_ to produce lentivirus. Infection of P3 and T98G cells was performed by centrifugation of cells with the virus at 2225 rpm for 90 min at room temperature in the presence of 10 μg/ml polybrene (Sigma-Aldrich). Enrichment selection using puromycin (1 μg/ml) (Sigma-Aldrich), followed by FACS-Sorting on Sony SH800 (Sony Biotechnology, San Jose, CA, United States) yielded stably expressing shRNA cells.

### Quantification of autophagic flux by CYTO-ID flow cytometry assay

Autophagic flux was induced by serum starvation through 3-hour culture in Earl´s balanced salt solution (EBSS) supplemented with 0.1% BSA). Inhibition of autophagic flux by blocking fusion between autophagosomes and lysosomes was induced by treatment with CQ (50 μM) for 24 h, final concentration of 50 µM for 3 h). Cells were treated with BTZ 10 nM for 48 h, with and without CQ 50 μM for 24 h. CYTO-ID Autophagy detection kit 2.0 (ENZ-KIT175-0050, Enzo Life Sciences, Farmingdale, NY, United States) was used to monitor the autophagic flux at the single-cell level as previously described (Lotsberg et al., 2020) and following manufacturer’s instructions. The dynamic autophagosome generation and clearance were assessed by live cell flow cytometry using BD Accuri C6 flow cytometer (BD Biosciences). Median fluorescence intensities (FL2-A channel) of 5000 cells are given. The dye of the Cyto-ID assay incorporates into autophagic vacuoles (pre-autophagosomes, autophagosomes, and autolysosomes (autophagolysosomes), and thus allows single-cell measurement of autophagic flux in lysosome inhibited live cells.

### Immunocytochemistry and confocal microscopy

Cells (4×10^4^ cells/well) were plated on coverslips in 24-well plates and subsequently treated with the various drug combinations as indicated above for 48 or 72 h. For LC3A/B and p62 (SQSTM1) and STX17 (Supplementary Table S1) staining, live cells were fixed with 3.7% formalin for 15 min at RT, stained with primary antibodies, followed by incubation with goat anti-rabbit-Alexa Fluor^®^555 and goat anti-mouse-Alexa Fluor^®^488 (Supplementary Table S1). Slides were mounted with Prolong Gold DAPI, and images were acquired on Leica TSC SP8 STED 3X (Leica).

### Transmission electron microscopy

Treated or control cells were fixed overnight in 2% glutaraldehyde in a cold medium and thereafter rinsed in 0.1 M Na-cacodylate buffer before post-fixation in 1% osmium (OsO_4_) in Na-cacodylate buffer for 1 h. Cells were washed again in 0.1 M Na-cacodylate buffer dehydrated in 30% ethanol for 20 min on ice, 50% ethanol for 20 min at 4°C, and finally, 70% ethanol overnight at RT. The second dehydration cycle was the following: 96% ethanol for 30 min at RT, 100% ethanol for 30 min (×2), and propylene oxide for 20 min. The sample was mixed in propylene oxide/agar 100 resin in a 1:1 ratio overnight at RT, and further hardened overnight at 60°C before sectioning to 45–50 nm. Imaging was performed on a Jeol JEM-1230 (Tokyo, Japan) at 80 kV using a gatan camera. The number of autophagosomes and autolysosomes were counted from at least 10 cells from two to three independent experiments by two independent investigators.

### RT-qPCR

RNA from P3 cells treated with BTZ alone, TMZ alone or combination with BTZ and TMZ were isolated using Qiagen RNeasy kit following the manufacturer’s instructions. cDNA was synthesized using the iScript cDNA Synthesis kit (BIO-RAD; Hercules, California, USA) according to the manufacturer’s instructions. iQ SYBR Green from the Supermix kit (BIO-RAD) was used to detect amplified produce in the PCR reaction mixture. The reaction was run on a Roche light cycler (LC480, Roche; Indianapolis, IN, United States) for 40 cycles. The primer sequences were as follows: ULK1 forward 5′- GGA​CAC​CAT​CAG​GCT​CTT​CC-3′ and reverse 5′- GAA​GCC​GAA​GTC​AGC​GAT​CT-3′, FOXO3a forward 5′- GCG​ACA​GCA​ACA​GCT​CTG​CC-3′ and reverse 5′- GGG​CTT​TTC​CGC​TCT​TCC​CCC-3′, PUMA forward 5′- TCA​ACG​CAC​AGT​ACG​AGC​G- 3′ and reverse 5′- AAG​GGC​AGG​AGT​CCC​ATG​AT -3′ and internal control 18S forward 5′-CGG​CTA​CCA​CAT​CCA​AGG​AA-3′ and reverse 5′-GCT​GGA​ATT​ACC​GCG​GCT -3’. Target transcripts were normalized to 18S and analyzed using the comparative CT (ΔΔCT) method.

### Intracranial implantation of glioblastoma spheroids

P3 wild type and P3 *ATG5* ^*−/−*^ GBM cells were propagated *in vitro* in the Neural Basal (NB) medium (Invitrogen, Hämeenlinna, Finland) supplemented with 1% GlutaMAX (Invitrogen, Carlsbad, CA, United States), 2% B-27 (Invitrogen), 5% penicillin–streptomycin, 10 mg/ml epidermal growth factor and fibroblast growth factor (PeproTech SE, Stockholm, Sweden) at 37°C in a humidified atmosphere of 5% CO2. For *in vivo* experiments, standardised neurospheres containing 10^4 ^cells/sphere were established by centrifuging cells in a CorningTM Clear Polystyrene V shaped-bottom 96-well plates in 0.5% v/v methylcellulose-NB for 2 h at 2250 rpm and 33°C. Spheroids were cultured for 1 week in the incubator at 37°C and 5% CO2, and media was changed at day 4. P3 and P3 *ATG5* ^*−/−*^ tumour spheroids (ten per animal) were implanted intracranially into male and female 7-week-old (20 g) severe combined immunodeficient (NOD-SCID) mice (C.B.-Igh-1b/lcrTac-Prkdc) (Janvier Labs; Le Genest-Saint-Isle, France) after anaesthetic with Servoflurane (Abbott Laboratories Ltd., Maidenhead, United Kingdom). After surgery, animals were allowed to recover from anaesthesia in a pre-warmed incubator and administered Temgesic (0.05 mg/kg, intraperitoneal injection, Indivior United Kingdom limited, Berkshire, United Kingdom) as analgesia for 3 days. Six animals were randomly assigned to each of the following treatment groups for both P3 wild type and P3 *ATG5* ^*−/−*^ implanted groups: 1) vehicle control; 2) BTZ monotherapy 0.5 mg/kg, human equivalent dose (HED), 1.3 mg/m^2^; 3) TMZ monotherapy with 50 mg/kg, HED, 164 mg/m^2^; 5) BTZ + TMZ 164 mg/m^2^ combination therapy. BTZ 1.3 mg/m2 was administered intraperitoneally on days 1, 4, 8, and 11, for two cycles with a 10-day break between cycles. TMZ chemotherapy was administered by oral gavage 5 days/week for 5 weeks, starting 48 h after BTZ treatment. The animals were housed in pairs in internally ventilated, closed cages (IVC) with standard bedding (NESTPAK™, Datesand, Manchester, United Kingdom). The animals were kept in an isolation facility at 25°C (55% relative humidity) on a standard 12 h night and day cycle in a specific pathogen-free environment and fed a standard pellet chow and provided tap water *ad libitum*. Animal husbandry protocols were followed where animals were monitored daily according to humane endpoint guidelines by experimental staff and independently, by animal husbandry staff, including inhouse veterinarian. Mice were sacrificed by CO_2_ inhalation and decapitation when neurological symptoms of rotational behaviours, reduced activity, grooming and or upon 20% body weight loss.

### Liquid chromatography mass spectrometry

The protein secretome in conditioned media of treated cells was analysed by Electrospray ionization LC-MS. Samples from two independent experiments were run on an Ultimate 3000 RSLC system (Thermo Scientific, Sunnyvale, California, United States) connected online to a Q-Excative HF mass spectrometer (Thermo Scientific, Bremen, Germany) equipped with EASY-spray nano-electrospray ion source source (Thermo Scientific). MS spectra (from m/z 375–1500) were acquired in the Orbitrap with resolution R = 60000 at m/z 200, automatic gain control (AGC) target of 3e6 and a maximum injection time (IT) of 110 ms. Complete methods are described in detail in supplementary methods.

### Mutational analysis of P3 and T98G GBM cells

Targeted sequencing of 360 cancer genes, was performed as previously described (Yates et al., 2015). Briefly, native, genomic DNA isolated from cell lines was fragmented and Illumina DNA sequencing libraries were prepared. Libraries were then hybridized to custom RNA baits according to the Agilent SureSelect protocol. Paired-end, 75 bp sequence reads were generated on an Illumina MiSeq instrument. Sequencing coverage for the targeted regions (average per bp) for the sample were 256x (P3) and 253x (T98G).

Raw mutations were called by the MiSeq Local Run Manager software and annotation was performed applying Annovar. Called variants were restricted to those passing all default filters and further filters were: including only coding variants, excluding synonymous variants, variant allele frequency > 0.1, population frequency <0.005 in 1K genomes project “all” and “eur”, population frequency <0.005 in esp6500.

### Statistical analysis

When comparing more than two groups with one dependent variable, one-way ANOVA was used, while two-way ANOVA was used to analyze data with two or more dependent variables compared in two or more groups. Bonferroni or Tukey’s *post hoc* correction for multiple testing was used. Descriptive statistics were reported as mean ± standard error of the mean (SEM) unless otherwise stated. Two-sided *p*-values less than .05 were considered significant (shown as *p < .05, **p < .01, ***p < .001, and ****p < .0001). All graphs represent the mean ± standard error of the mean (S.E.M.) of at least 3 independent experiments. All statistical analyses were performed in Stata version 13.1 (Texas, United States) or GraphPad Prism v6.07 (La Jolla, CA, United States).

## Results

### Bortezomib synergistically enhances the sensitivity of GBM cells to temozolomide chemotherapy

**FIGURE 1:**
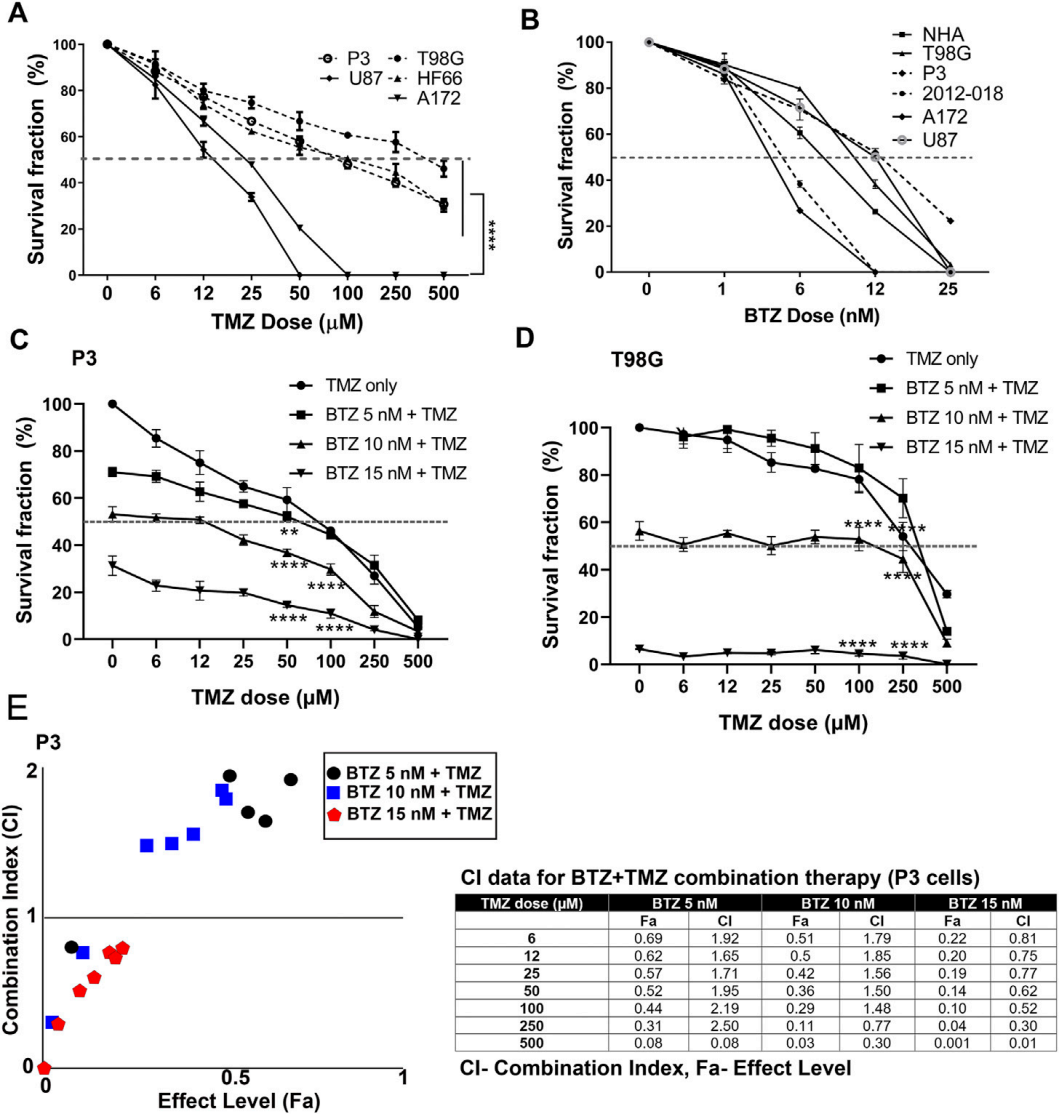
BTZ pre-treatment synergizes with TMZ treatment and chemosensitizes GBM cells **(A)** Mean % ± S.E.M clonogenic survival of tumour cells treated with TMZ for 72 h **(B)** Mean % ± S.E.M clonogenic survival of tumour cells and normal human astrocytes (NHA) treated with BTZ for 48h. Clonogenic surviving fractions of **(C)** P3 and **(D)** T98G tumour cells after specified treatments, BTZ concentration was 0, 5, 10 and 15 nM, respectively, and TMZ concentration in 6–500 μM range. **(E)** Combination index plot (left) and data table (right) from report generated by Compusyn software for drug combination synergy calculation following the Chou-Talalay method of P3 cells treated with BTZ 5, 10, and 15 nM and TMZ concentration in 6–500 μM range. Combination index (CI) quantitatively determine “Synergism (CI < 1), additive-effect (CI = 1) and antagonism (CI > 1). Each experiment was performed in triplicate, and data represents the mean ± S.E.M of at least 3 independent experiments, **p < .01 and ****p < .0001.

We first investigated the clonogenic survival of a panel of patient derived GBM cells and cell lines in response to various doses of TMZ and BTZ alone or in combination. P3, T98G, and HF66 GBM cells were most resistant to TMZ, requiring higher doses (>50 μM) to kill 50% of the cells, compared to U87 and A172 GBM cells whose IC_50_ doses were 12 and 25 μM, respectively (*p* = .0001, Figure 1A). In contrast, all GBM cell lines, as well as control cells (immortalized normal human astrocytes (NHA)) were sensitive to low doses of BTZ (IC_50_ range 6–12 nM, Figure 1B). Pre-treatment with 5 nM, 10 nM and 15 nM BTZ prior to TMZ effectively reduced the clonogenic survival of P3 and T98G cells compared to TMZ treatment alone. BTZ pre-treatment potentiated the effect of all TMZ doses for both P3 and T98G cells (*p* < .0001, Figures 1C,D, respectively), where 15 nM BTZ was synergistic with all TMZ doses (Figure 1E). However, the concentration of 500 μM TMZ treatment was synergistic with 5 nM BTZ and 250 μM and 500 μM TMZ treatment were synergistic with 10 nM BTZ. Taken together, pre-treatment with BTZ rescinded GBM cells’ resistance to TMZ.

### Genetic background of the GBM cells

To rule out that differences in genetic mutations accounted solely for the differential responses to BTZ and TMZ treatment, we characterized the genetic background of P3 and T98G cells, Table 1. Both cell types harbored wild-type (Wt) isocitrate dehydrogenase 1 gene (*IDH1*) that denoted them as *de novo* GBM. P3 cells harbored a homozygous *Cys176Tyr* nonsynonymous single nucleotide variant (SNV) in the tumour protein 53 (*TP53*) gene, whilst T98G cells possessed a *Met237Ile* SNV mutation in *TP53*. Both P3 and T98G cells had mutations in *MAP3K1* and in the phosphatase tensin homolog (*PTEN*) gene, while additionally, P3 cells harbored a heterozygous *PIK3R2* mutation in exon 11 and in *STAT3*, potentially inducing aberrant PI3K signaling (Table 1). Particularly in P3 cells, these aberrations are implicated in the development of autophagy addiction (Guo et al., 2011).

**TABLE 1:** Genetic background of GBM cell lines.

	P3	T98G
TP53	Mut C176Y (1/1)	Mut M237I (1/1)
PTEN	Mut L220fs (0/1)	Mut L42R (1/1)
PIK3R2	Mut Q445H (0/1)	Wt
BRAF V600E	Wt	Wt
IDH1	Wt	Wt
EGFR	Wt	Wt
MAP3K1	Mut (0/1) nonframeshift deletion exon14:c.2822_2824del:p.941_942del	Mut D1170Y (0/1) nonframeshift deletion exon14:c.2822_2824del:p.941_942del
STAT3	Mut H131R (0/1)	Wt

Genotype (GT): 0/1 (heterozygous, carrying 1 copy of each of the reference and alternate allele), and 1/1 (homozygous alternate allele), Mut: Mutation, Wt: Wild type.

### BTZ and BTZ + TMZ combination treatment induce an accumulation of lipidated LC3A/B (LC3A/B-II) and p62 (SQSTM1) in patient derived GBM cells

**FIGURE 2:**
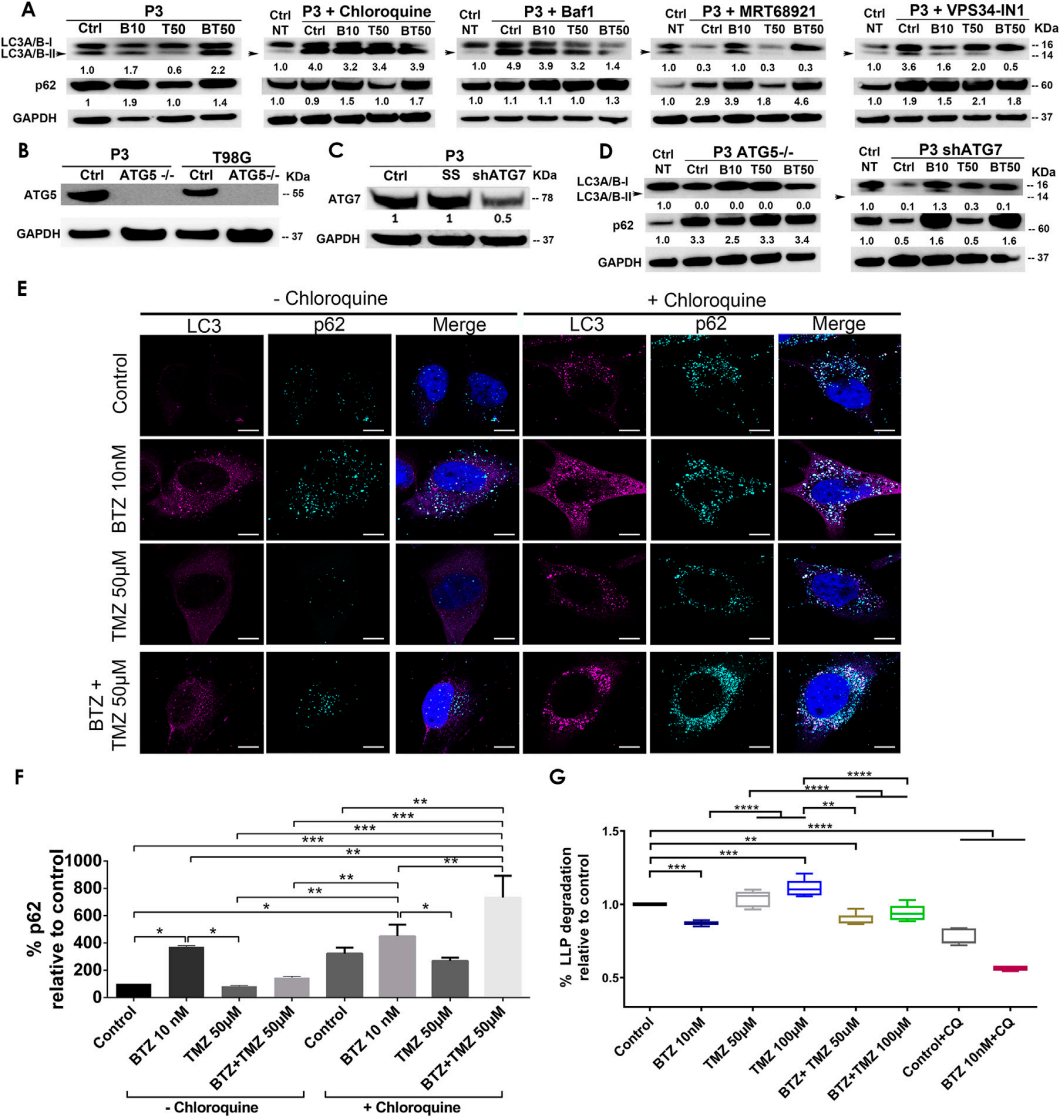
BTZ and combination BTZ + TMZ treatment accumulates LC3A/B-II and p62 (SQSTM1) in patient derived GBM cells. **(A)** Western blot of the indicated proteins in lysates of wild type P3 cells and P3 cells treated with 10 nM BTZ (B10), 50 μM TMZ (T50) or both (BT50) or in a combination with autophagy inhibitors (chloroquine, Bafilomycin A1 (BafA1), MRT68921 or VPS34-IN1). **(B,C)** Western blot of the indicated proteins in lysates of wild type P3, T98G cells and cells lacking *ATG5* (*ATG5* ^*−/−*^)*,* scrambled shRNA (SS) and ATG7 (shATG7). **(D)** Western blot of the indicated proteins in lysates of wild type P3 cells lacking *ATG5* (*ATG5* ^*−/−*^) or ATG7 (shATG7), treated with 10 nM BTZ (B10), 50 μM TMZ (T50) or both (BT50). GAPDH was used as a loading control and for normalizing densitometry measurements of the blots. Ctrl NT = non-treated control cells. **(E)** Confocal microscopy of P3 cells stained with antibodies against LC3A/B and p62 (SQSTM1) after the indicated treatments in the absence (left panel) or presence (right panel) of chloroquine (CQ), scale bar 10 μm. **(F)** Quantification of p62 (SQSTM1) puncta after the indicated treatments in the absence or presence of CQ autophagy inhibition. Data represents % mean ± S.E.M from at least 10 cells from two to three independent experiments. **(G)** Percent degradation of long-lived proteins (LLPD) relative to untreated control, quantified as release of ^14^C-valine after indicated treatments in the presence or absence of CQ. *p < .05, **p < .01, ***p < .001 and ****p < .0001.

Autophagy is frequently deregulated in cancer and is considered a maladaptive process, promoting both cancer cell survival or death at different stages of disease progression or in response to treatment (Kanzawa et al., 2004; Sui et al., 2013), in a cell-specific manner and in response to proteasome inhibitors. Since TMZ monotherapy was unable to efficiently kill GBM cells, we hypothesized that increased autophagic flux might have sustained the survival of GBM cells during TMZ treatment. The levels of the autophagic cargo receptor p62 (SQSTM1) in protein lysates from P3 cells after TMZ treatment were minimally altered relative to untreated controls, whereas the level of the autophagy marker LC3A/B-II was diminished by approximately 40% (Figure 2A), potentially indicating sustained autophagy. In contrast, treatment with 10 nM BTZ induced accumulation of both LC3A/B-II and p62 (SQSTM1) protein levels by approximately 2-fold after 48 h (Figure 2A). However, 15 nM BTZ increased LC3A/B-II and p62 (SQSTM1) protein levels by 14 and 8-fold, respectively, relative to untreated controls (Supplementary Figure S1). Combining 10 nM BTZ with different TMZ doses and time points resulted in increased levels of LC3A/B-II (approximately 2-fold) and p62 (SQSTM1) levels approximately 2-fold (Supplementary Figure S1), potentially indicating accumulated autophagosomes. In T98G cells, LC3A/B-II levels were only induced by combination 10 nM BTZ + 250 μM TMZ as well as chloroquine (CQ) treatment with modest changes in p62 (SQSTM1) (Supplementary Figure S2A) potentially indicating reduced susceptibility in autophagosome accumulation in these cells.

### BTZ and BTZ + TMZ mediated accumulation of LC3A/B-II and p62 (SQSTM1) autophagosomes in GBM cells is dependent on core autophagy machinery

To investigate whether the increase in LC3A/B-II and p62 (SQSTM1) protein levels in BTZ and BTZ + TMZ treated cells were autophagy-specific and identify the particular stage affected in the multistep process, we targeted ULK1/2 and PIK3C3/VPS34 proteins that are both involved in early phagophore initiation. Both MRT68921 and VPS34-IN1, inhibitors of ULK1/2 and VPS34 respectively, prevented LC3A/B lipidation (Figure 2A). 2-Fold elevated p62 (SQSTM1) levels were detected in cells co-treated with BTZ and these inhibitors. The late stage autophagy inhibitors Bafilomycin A1 (BafA1) and CQ both inhibit autophagosome-lysosome fusion by affecting acidification (Yamamoto et al., 1998), and like BTZ, they also induced approximately 4-fold elevation of LC3A/B-II levels in the absence or presence of BTZ (Figure 2A). Elongation and expansion of the phagophore is enabled by conjugation of LC3A/B-II to phosphatidylethanolamine (PE), facilitated by the E1 ligase ATG7 and the E3-like complex ATG12-ATG5-ATG16L1. Targeting *ATG5* in this conjugation complex by CRISPR-Cas9 completely abolished ATG5 protein (Figure 2B) whereas small hairpin RNA mediated knockdown of ATG7 mRNA (ATG7 shRNA) depleted 50% of the protein (Figure 2C). *ATG5* ^−/−^ and ATG7 shRNA cells completely lacked lipidated LC3A/B and the p62 (SQSTM1) level was upregulated by approximately 3-fold in *ATG5* ^*−/−*^ cells, but not after BTZ treatment (Figure 2D). In contrast, BTZ treated ATG7 shRNA cells were still able to upregulate p62 (SQSTM1) by approximately 2-fold (Figure 2D).

In line with these western blot data, immunofluorescence microscopy revealed that LC3A/B and p62 (SQSTM1) puncta increased after treating P3 cells with BTZ alone or in combination with TMZ, but not under steady state or when treated with TMZ alone (*p* < .0001, *p* < .05, and *p* > 0.05, respectively, Figures 2E,F). p62 (SQSTM1) positive puncta were also prominently observed in the presence of the lysosomal inhibitor CQ in P3 cells (Figures 2E,F) suggesting that BTZ leads to accumulation of autophagosomes, as does the late-stage inhibitors, BafA1 and CQ.

### Increased autophagosome accumulation after BTZ and BTZ + TMZ treatment is due to block in autophagic flux as a result of diminished autophagosome-lysosome fusion

To confirm that increased autophagosome formation after BTZ treatment was due to abrogated cargo degradation and not increased autophagic flux, we assessed the effect of the BTZ treatment on the degradation of long-lived proteins known to be degraded by autophagy. Cells were pulsed with radiolabeled valine, and following a chase to deplete short-lived proteins and induction of autophagy by starvation, the long-lived protein degradation (LLPD) was quantified (Figure 2G). Indeed, the level of recycled amino acids was higher after treatment with 100 μM TMZ in P3 cells compared to control (*p* < .001) and in TMZ 100 μM *vs*. BTZ + TMZ 100 μM (*p* < .0001, Figure 2G). In contrast, BTZ monotherapy significantly reduced the degradation of long-lived proteins compared to untreated control (*p* < .0001) and both TMZ 50 μM and 100 μM (*p* < .0001, Figure 2G), consistent with an abrogated cargo degradation, *ergo* abrogated autophagic flux. BTZ + TMZ 50 μM also attenuated the degradation of long-lived proteins compared to controls and TMZ 50 μM (*p* < .01 respectively, Figure 2G) and confirmed the inhibition of autophagic flux by blocking degradation of cargo. As expected, the autophagy inhibitor CQ inhibited autophagic flux in all treatment conditions (*p* < .0001, Figure 2G), similarly to BTZ treatments. These findings were corroborated in T98G cells, where BTZ containing treatment regimen also attenuated degradation of long-lived proteins compared to untreated controls or TMZ chemotherapy (*p* < .0001, Supplementary Figure S2B).

**FIGURE 3:**
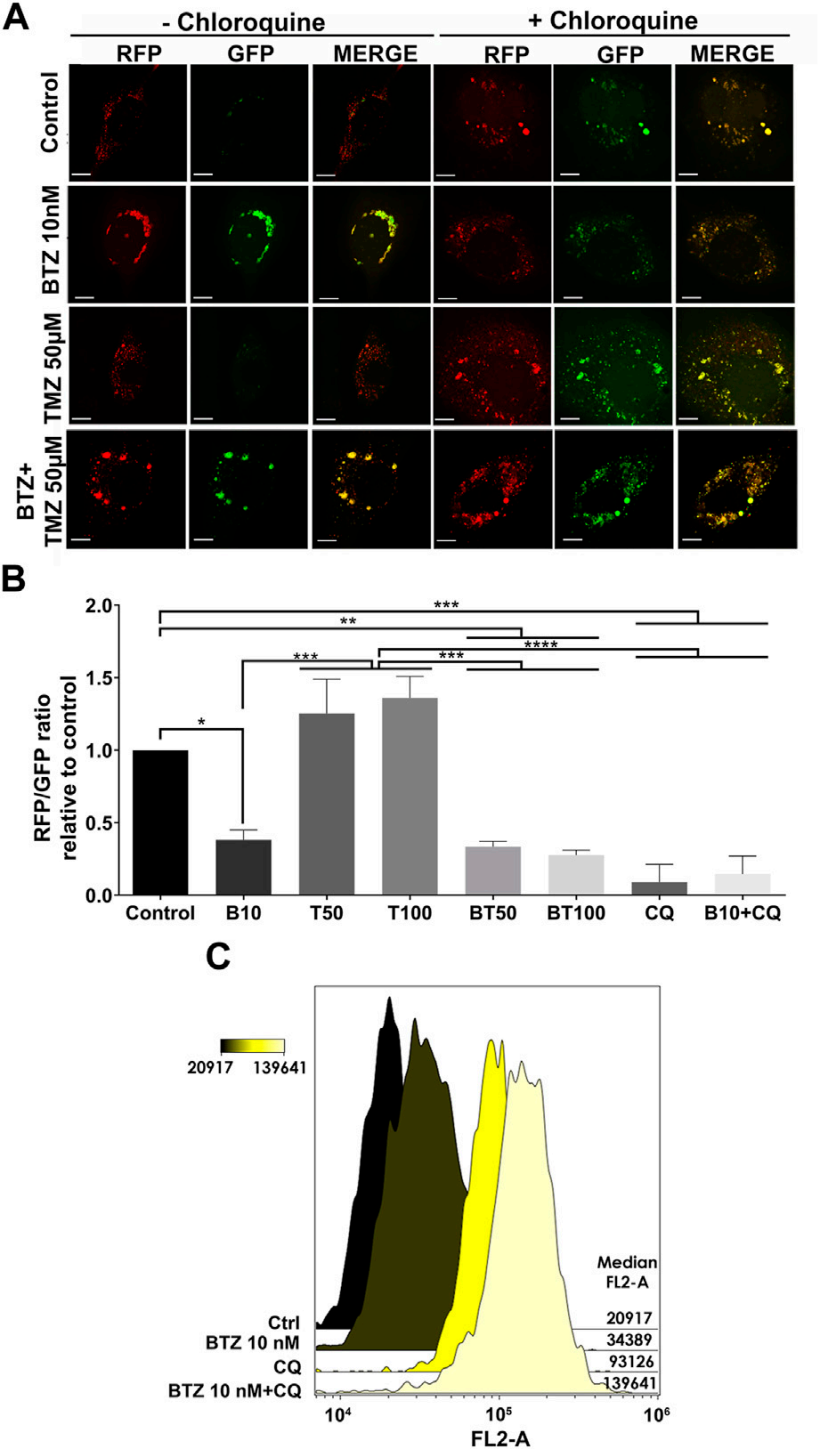
LC3A/B+ autophagosomes accumulate in response to BTZ and combination BTZ + TMZ treatment of GBM cells **(A)** Confocal microscopy of P3 cells transfected with RFP-GFP-LC3B tandem probe and subsequently treated as indicated, red (RFP) fluorescence marks autolysosomes and yellow (merge of RFP and GFP) autophagosome puncta formation in the presence or absence of CQ, scale bar 10 μm. **(B)** Quantification of RFP puncta relative to GFP puncta in cells treated with 10 nM BTZ (B10), 50 μM TMZ (T50), 100 μM TMZ (T100) or both (BT50 and BT100) in the absence or presence of CQ, normalized to untreated control cells. *p < .05, **p < .01, ***p < .001 and ****p < .0001. **(C)** Autophagic flux under 3 h starvation in EBSS medium of P3 cells was assessed by CytoID probe following pre-treatment as indicated (BTZ 10 nM 48h, Chloroquine (CQ) 50 μM 24 h). Histograms are representative of three independent experiments. The color scale represents median fluorescence intensity (MFI), and values for each treatment are shown on the right side of the histograms.

As a further confirmation that BTZ alone and in BTZ + TMZ combination treatment abrogated autophagic flux, we used the Autophagy Tandem Sensor RFP-GFP-LC3B probe to dynamically monitor the formation of autophagosomes vs*.* autolysosomes over time in the presence or absence of CQ. Both untreated controls and TMZ monotherapy treated cells predominantly displayed red-only puncta, representing degradative autolysosomes, indicating that autophagic flux was retained during TMZ treatment (Figures 3A, B). In contrast, BTZ treatment alone or BTZ + TMZ combination treatment blocked autophagic flux, as indicated by a 60%–70% increase of yellow puncta (ratio of green vs*.* red puncta) autophagosomes relative to untreated control cells (*p* < .05) as well as BTZ alone compared to TMZ 50 μM (*p* < .01); TMZ 100 μM (*p* < .001) or BTZ + TMZ 50 μM and BTZ + TMZ 100 μM, *p* < .001 for both, Figures 3A, B). As expected, addition of CQ increased the frequency of yellow puncta, representing autophagosomes, under all treatment conditions (Figures 3A,B).

Autophagic flux at the single-cell level was quantified by flow cytometry using the Cyto-ID probe, which stains autophagic vesicles. When autophagic flux was abrogated by BTZ treatment in nutrient-deprived cells, relative median fluorescence intensity was increased by approximately 2-fold relative to untreated controls (Figure 3C). Likewise, treatment with CQ strongly induced the accumulation of autophagic vesicles, as indicated by 4-fold increase in median fluorescence intensity relative to control cells. Addition of CQ to the BTZ pre-treated cells had an additive effect and approximately 6-fold increase in median fluorescence intensity (Figure 3C). Taken together, these data indicate that BTZ treatment abrogates autophagic flux by blocking fusion of autophagosomes with lysosomes under both nutrient-rich and nutrient-deprived conditions.

### BTZ abrogates autophagic flux by blocking autophagosome-lysosome fusion in an ATG5 dependent manner

**FIGURE 4:**
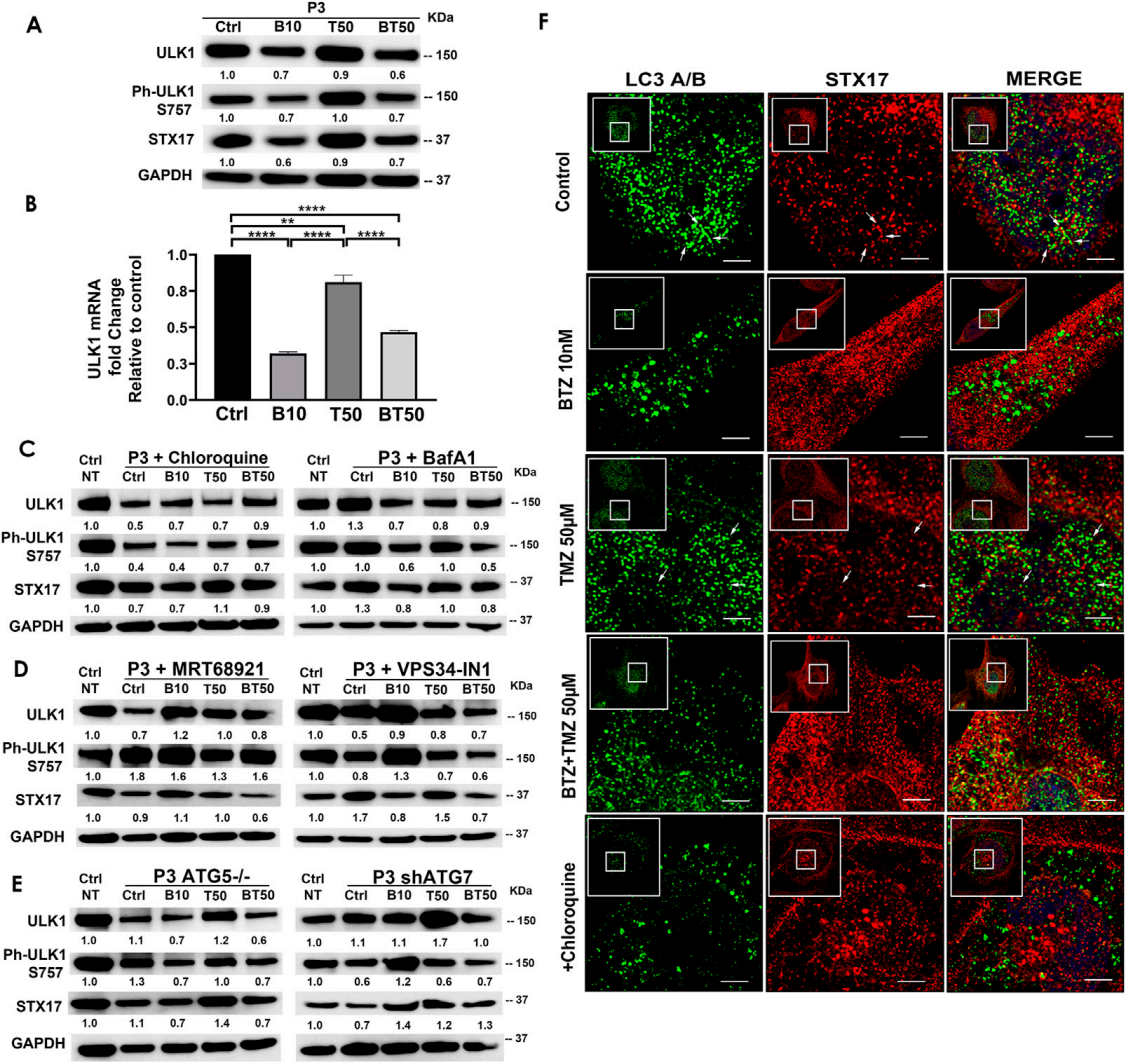
BTZ abrogates autophagic flux by blocking autophagosome-lysosome fusion. **(A)** Western blot of the indicated proteins in lysates of wild type P3 cells treated with 10 nM BTZ (B10), 50 μM TMZ (T50) or both (BT50) **(B)** ULK1 mRNA fold change after indicated treatments in P3 cells. **(C,D)** Western blot of the indicated proteins in lysates of P3 cells treated with 10 nM BTZ (B10), 50 μM TMZ (T50) or both (BT50) or in a combination with autophagy inhibitors (chloroquine, BafA1, MRT68921 or VPS34-IN1) **(E)** Western blot of the indicated proteins in lysates of P3 cells lacking *ATG5* (*ATG5* ^*−/−*^) and ATG7 (shATG7). GAPDH was used as a loading control and for normalizing densitometry measurements of the blots. GAPDH blots in C is from the same samples/experiments used in Figure 2. Ctrl NT = non-treated control cells. **(F)** Confocal microscopy of P3 cells co-stained for LC3A/B and STX17, subsequently treated as indicated, in the presence or absence of CQ, scale bar 5 μm; for the zoomed part of the image from the image shown in the white square. White arrows are indicating the STX17 and LC3A/B colocalization.

**FIGURE 5:**
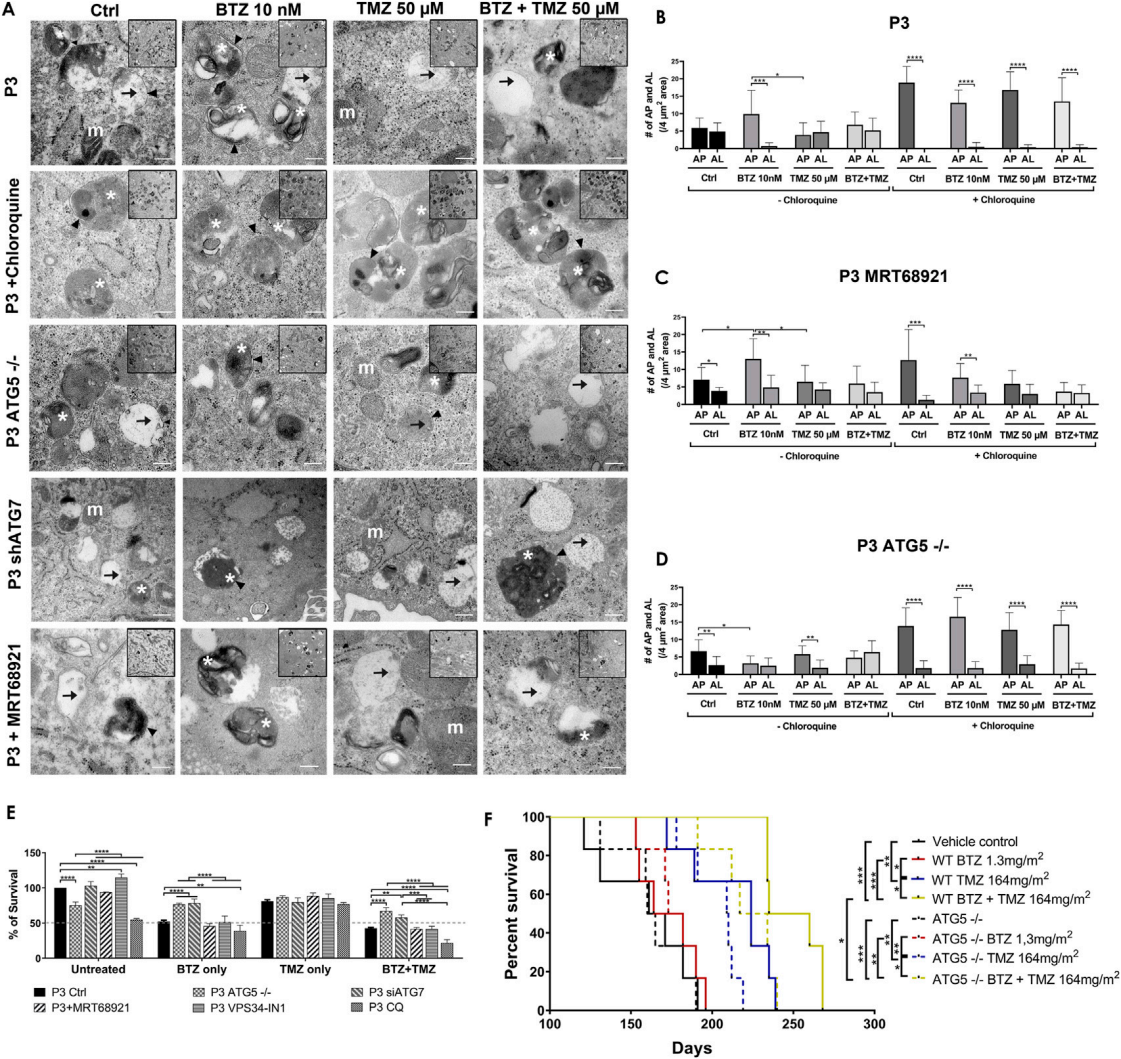
Targeting autophagy diminishes tumour cell survival **(A)** Transmission electron microscopy of untreated, BTZ or TMZ monotherapy and BTZ + TMZ combination treated P3 ctrl cells, *ATG5* ^*−/−*^ P3 cells and ATG7 knockdown P3 cells as well as P3 cells treated with chloroquine and MRT68921, scale bar 200 nm, magnification 50K. Inserts 20K magnification. Arrowhead = . double membrane, white asterix = autophagosomes, arrow = autolysosomes and m (white) = mitochondria. **(B–D)** The number of autophagosomes and autolysosomes were counted from at least 10 cells from two to three independent experiments by two independent investigators in P3 control cells, P3 cells treated with MRT68921 and P3 cells lacking *ATG5* (*ATG5* ^*−/−*^) with or without CQ. **(E)** Clonogenic survival of untreated or treated P3 control, *ATG5* ^*−/−*^, shATG7, MRT68921, VPS34-IN1and CQ treated cells after experimental treatments. **(F)** Kaplan–Meier curves showing percentage survival in days. Solid lines representing P3 wild-type cells and dashed lines representing P3 *ATG5* ^*−/−*^ cells implanted mice percentage survival with indicated treatments. *p < .05, **p < .01, ***p < .001 and ****p < .0001.

Confusion exists regarding whether proteasome inhibitors augment (Zhu et al., 2010; Armstrong et al., 2011) or prohibit autophagic flux (Periyasamy-Thandavan et al., 2010; Kao et al., 2014), largely due to the lack of use of proper flux assays and misinterpretations of LC3A/B immunoblotting as a surrogate marker for flux (Mizushima and Yoshimori, 2007). As proof of concept, we targeted the five stages of autophagy: phagophore initiation/nucleation (UKL1/2 and VPS34 inhibitors), expansion and elongation (ATG5 and ATG7 depletion), autophagosome closure and lysosomal fusion (bafilomycin A1 and chloroquine) and examined changes in expression of ULK1/2, STX17, LC3A/B-II, and p62 (SQSTM1) before and after 48 h treatment with BTZ by immunofluorescence, subcellular morphology, western blot of cell lysates, as well as cell clonogenic survival (Figure 4 and Figure 5).

P3 GBM cells treated with BTZ or BTZ + TMZ had 30%–40% attenuated total and phosphorylated ULK1 expression (Figure 4A), which was *not* restored in the presence of CQ, and as reflected in 60%–70% reduction in mRNA levels compared to control and TMZ treated cells (*p* < .0001, respectively, Figure 4B). In contrast, ULK1 levels in TMZ treated cells were more similar to untreated controls (*p* < .01). BTZ and BTZ + TMZ treated P3 cells diminished expression of STX17 marker for lysosomes by 30%–40% in presence or not of CQ (Figures 4A–C). Targeting ULK1 and VPS34 protein complexes involved in phagophore nucleation with MRT68921 (UKL1/2 dual inhibitor) and VPS34-IN1 depleted lipidated LC3A/B treatment (Figure 2A), although in presence of VPS34-IN1, STX17 expression was slightly reduced (Figure 4D). Targeting *ATG5* during BTZ treatment had no major impact on phospho-ULK1 or STX17 expression, but ATG7 shRNA cells treated with TMZ increased STX17 (Figure 4E). Immunofluorescence staining for STX17 and LC3A/B on treated cells revealed absence of STX17/LC3A/B colocalization in BTZ or CQ treated cells but colocalization was evident in untreated controls and TMZ treated cells, (Figure 4F). Taken together, the data indicate that BTZ prevents ULK1 phosphorylation and leads to accumulation of LC3A/B-II in an *ATG5* dependent manner in the early stages, subsequently abrogating flux by blocking autophagosome-lysosome fusion, indicated by low levels of STX17 colocalization with LC3A/B.

Transmission electron microscopy confirmed the equal presence of autophagosomes as well as fused degradative autolysosomes in control and TMZ 50 μM treated cells (Figures 5A,B), but 5-fold increase in double-membrane limited autophagosomes containing preserved organelles in BTZ treated cells compared to autolysosomes (*p* < .001, Figure 5B) and compared to TMZ 50 μM treated cells (*p* < .05). As expected, autophagosomes were significantly increased compared to autolysosomes regardless of treatment (*p* < .0001) in the presence of CQ. Corroborating the observed increases in lipidated LC3A/B, inhibition of ULK1/2 with MTR68921 in control and BTZ treated cells significantly increased the frequency of autophagosomes relative to autolysosomes (*p* < .05 and *p* < .01, respectively), that were also *not* further augmented in the presence of CQ (*p* < .001 and *p* < .01, respectively), and compared to control and TMZ 50 μM treated cells, (*p* < .05 respectively, Figures 5A,C). In the *ATG5* knockout cells, only control and TMZ treated cells displayed an increased ratio of autophagosomes relative to autolysosomes, (*p* < .01), wherein these *ATG5* ^*−/−*^ cells, BTZ treatment failed to induce autophagosomes (*p* < .05, Figures 5A,D). As expected, in the presence of CQ autophagosomes were increased in all treatment conditions (*p* < .0001), and when effect of BTZ was abrogated by CRISPR-Cas9 *ATG5* ^*−/−*^ (Figures 5A,D).

### Diminished tumour cell survival and reduced animal survival due to BTZ abrogated autophagic flux requires a functional autophagy machinery

To be certain that BTZ mediated abrogation of autophagic flux was responsible for the biological effects on GBM cell death during combination treatment with TMZ, colony formation as an indication of clonogenic survival was monitored in P3 and T98G cells depleted of *ATG5* or ATG7, or treated with MRT68921 (ULK1/2 inhibitor) or VPS34-IN1 (VPS34 inhibitor) under steady state and during BTZ and TMZ treatment. CRISPR-Cas9 ablation of *ATG5* in both P3 and T98G cells reduced clonogenic survival compared to wild type cells (*p* < .0001 and *p* < .01, respectively) and compared to wild type cells treated with MRT68921, VPS34-IN1 to inhibit phagophore initiation (early step), or chloroquine to inhibit lysosomal acidification (late step) (*p* < .0001, Figure 5E and Supplementary Figure S2C). ATG7 shRNA did not affect cell survival of neither P3 nor T98G cells. However, while BTZ treatment reduced P3 cell survival by 50% and could not be further augmented by addition of MRT68921 or VPS34-IN1, the cytotoxic efficacy of BTZ was annihilated by *ATG5* and *ATG7* depletion in P3 cells, which lacked LC3A/B-II and failed to accumulate autophagosomes compared BTZ treated controls (*p* < .0001 respectively, Figure 5E). Neither *ATG5* nor ATG7 depletion could rescue T98G cells from BTZ induced cytotoxicity (Supplementary Figure S2C). Addition of chloroquine enhanced BTZ cytotoxicity in P3 wild type cells. Clonogenic survival of *ATG5* ^−/−^ and ATG7 shRNA cells in response to TMZ treatment was not affected, nor when ULK1/2 or VPS34 was blocked with MRT68921 or VPS34-IN1 autophagy inhibitors in P3 cells (Figure 5E). In contrast, TMZ effect was marginally improved by addition of VPS34-IN1 in T98G cells (*p* < .05, Supplementary Figure S2C). However, survival was augmented in both P3 *ATG5* ^−/−^ and ATG7 knockdown cells (*p* < .0001 and *p* < .01 respectively, Figure 5E) and in only T98G *ATG5* ^−/−^ cells (*p* < .0001, Supplementary Figure S2C) in response to BTZ + TMZ treatment relative to controls. The BTZ cytotoxicity is diminished in both *ATG5,* and ATG7-depleted P3 cells, while in T98G cells, the BTZ effect was only apparent in *ATG5* ^*−/−*^ ablated T98G cells treated with a combination BTZ + TMZ. In contrast, attenuated LC3A/B-II formation through inhibiting ULK1/2 or VPS34 complexes was also reflected in the differential killing efficacy of P3 and T98G GBM cells, as was treatment with BTZ alone.

To investigate the diminished cytotoxicity of BTZ during combination treatment with TMZ in ATG5 knock-out P3 cells *in vivo*, NOD-SCID mice were orthotopically implanted with P3 wild type and ATG5 knock-out cells to generate xenograft models. The mice were treated intraperitoneally with 1.3 mg/m^2^ BTZ on days 1, 4, 8, and 11 for two cycles with a 10-day break between cycles. TMZ chemotherapy (164 mg/m^2^) was administered by oral gavage 5 days/week for 5 weeks, and the TMZ treatment was started 48 h after the first BTZ treatment in the combination treatment group mice. The vehicle control and BTZ monotherapy treated groups mice died earlier than the TMZ and combination-treated groups in both P3 wild type and *ATG5* ^*−/−*^ cells implanted mice within 190 days post-implantation. In P3 wild-type cells implanted groups, the median survival of the vehicle control group was 166 days compared to TMZ treated group with 224 days (*p* < .01) and to BTZ + TMZ combination group with 247 days (*p* < .001) (Figure 5F). The median survival was improved with 23 days in BTZ + TMZ combination group compared to TMZ monotherapy group (*p* < .05). In *ATG5* ^*−/−*^ cells implanted groups, the median survival of the vehicle control group was 162 days compared to TMZ treated group with 209 days (*p* < .01) and to BTZ + TMZ combination group with 225 days (*p* < .001) (Figure 5F). In the BTZ + TMZ combination-treated groups, the mice implanted with *ATG5* ^*−/−*^ cells have a shorter median survival of 225 days compare to the mice implanted with P3 wild type cells with 247 days (*p* < .05). These data suggest that BTZ + TMZ combination treatment prolonged animal survival, but BTZ mediated cytotoxicity to GBM cells requires a functional autophagy machinery that lacks in *ATG5* ^*−/−*^ cells.

### Autophagy abrogation increased MAP1LC3B/p62 secretion in ATG5 ^−/−^ P3 cells, as well as after BTZ and BTZ + TMZ treatment

Untreated *ATG5* ^*−/−*^ P3 cells accumulated more autophagosomes than autolysosomes (*p* < .01) also compared to BTZ treated cells (*p* < .05, Figure 5D), yet lysates contained undetectable LC3A/B-II protein. To investigate whether these autophagosomes might have been expelled to the extracellular matrix, we investigated the cellular secretome during nutrient-deprivation by liquid chromatography mass spectrometry. In total 3720 proteins were identified, where 2735 proteins were used for quantification. Hierarchical clustering from 164 statistically significantly secreted proteins identified 7 distinct biological processes involved in proteasome function, lysosomal metabolic processes, protein degradation and autophagy (Supplementary Figure S3). P3 *ATG5* ^*−/−*^ cells or P3 wild type cells treated with BTZ or BTZ + TMZ combination showed similar clustering in secretory autophagy and mitophagy related proteins compared to supernatants from control and TMZ treated cells that clustered more closely together (Supplementary Figure S3A). More MAP1LC3B and p62 (SQSTM1) were secreted into the supernatants in *ATG5* ^*−/−*^ P3 cells or when autophagy flux was abrogated with BTZ or BTZ + TMZ treatment compared to TMZ only or untreated controls (*p* < .001 and *p* = .007, Supplementary Figure S3B). Taken together, our data indicate that BTZ kills GBM cells by inhibiting autophagic flux, prohibiting autophagosome-lysosome fusion and subsequent degradation of cargo.

### BTZ induces DNA damage signalling and cell cycle arrest in TMZ treated GBM cells

**FIGURE 6:**
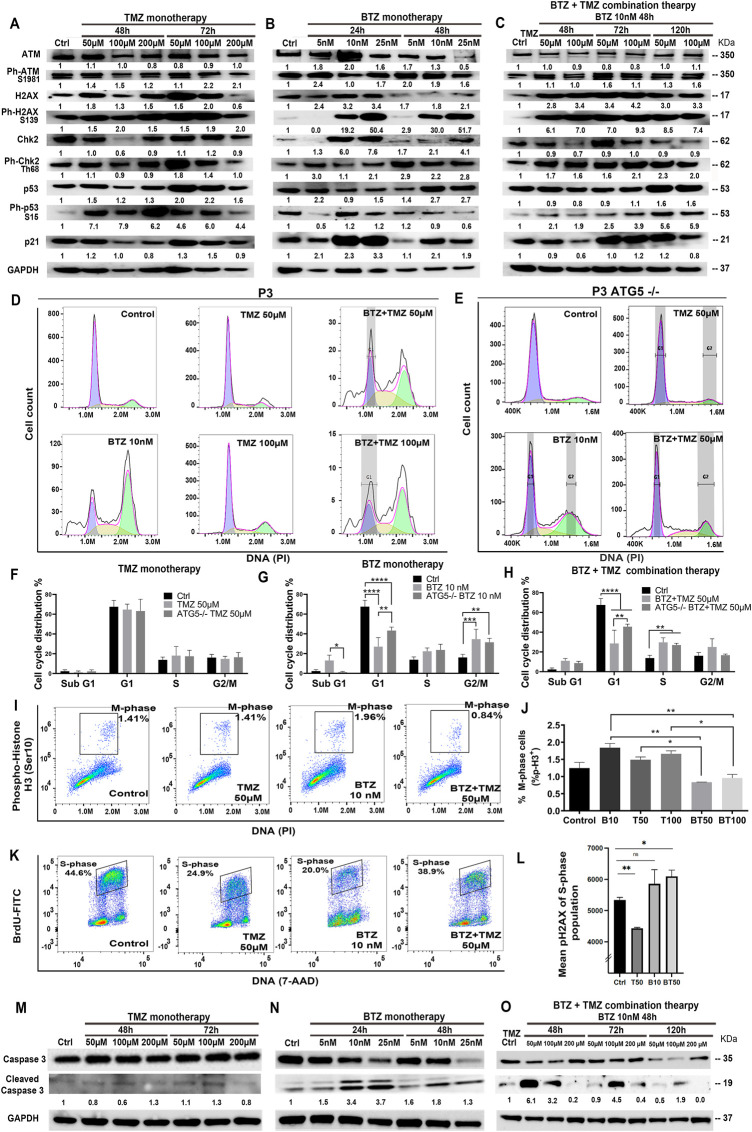
BTZ pre-treatment induces DNA damage signalling and G2/M phase cell cycle arrest in TMZ treated patient derived GBM cells. Proteins in DNA damage signalling cascade including total and phospho-proteins from lysates (20 µg) from P3 GBM cells treated with **(A)** TMZ monotherapy, dose escalation at 48 and 72h, **(B)** BTZ monotherapy, dose escalation at 24 and 48h and **(C)** combination treatment with BTZ 10 nM 48h and TMZ at indicated times. **(D,E)** DNA histograms showing cell cycle distribution of propidium iodide (PI) stained control and *ATG5* ^*−/−*^ P3 cells, before and after respective treatments. Quantification of cellular proportions (%) in respective cell cycle phases after **(F)** TMZ monotherapy, **(G)** BTZ monotherapy and **(H)** BTZ + TMZ therapy. **(I)** Dot plots of P3 cells in M-phase (phospho-histone H3 positive cells) after the respective treatments; **(J)** Quantification of experiments are shown in panel I, showing % of P3 cells in M-phase of the cell cycle as indicated. **(K,L)** Evaluation of treatment-induced DNA double-strand breaks (DSBs) by flow cytometry. Immunofluorescent staining of pH2AX and BrdU was performed as described in treated and BrdU pulsed cells to determine the level DSBs in S-phase (BrdU incorporating) populations by flow cytometry. **(K)** Representative dot plots showing BrdU incorporation *versus* DNA **(L)** Mean pH2AX expression + -SD of S-phase population of the various experimental conditions are shown. **(M–O)** Western blot analysis of total and cleaved caspase 3 in proteins lysates from P3 GBM cells treated with **(M)** TMZ monotherapy, **(N)** BTZ monotherapy and **(O)** combination treatment with BTZ + TMZ, at indicated times. GAPDH was used as a loading control and for normalizing densitometry measurements of the blots. *p < .05, **p < .01, ***p < .001 and ****p < .0001.

Since clonogenic survival after TMZ treatment appeared to be sustained by autophagic flux, and BTZ alone or in combination with TMZ promoted GBM cell death, we sought to investigate the crosstalk between abrogated autophagic flux and cell death, as well as characterize the mechanism of death. First, we examined effects of the treatments on DNA damage and perturbation of cell cycle kinetics. Thus, we investigated the phosphorylation status of the DNA damage sensors ATM, H2AX and Chk2 in chemoresistant GBM cells (P3 and T98G) in response to BTZ and TMZ mono- or combination therapy. At the IC_50_ dose of either TMZ or BTZ alone, the phosphorylation of serine and threonine residues of ATM (Ser 1981), H2AX (Ser139) and Chk2 (Thr68) was consistent with initiation of DNA damage signalling cascade at 48 h in P3 cells (Figures 6A, B). These changes in protein phosphorylation and downstream mediators were delayed in response to TMZ monotherapy compared to BTZ monotherapy (Figures 6A,B). BTZ monotherapy induced p53 and p21 expression within 24 h compared to TMZ monotherapy, where increases in phosphorylated ATM, H2AX, and p53 (Ser15), as well as the downstream genes Chk2 (3-fold) and p21 was 2-fold upregulated after 48 h (Figures 6A,B). Treatment of P3 cells with a combination of TMZ and BTZ led to 2-3-fold increased phosphorylation of ATM, H2AX and p53 after 48 h, but with half the TMZ IC_50_ dose (Figure 6C), implying augmented DNA damage response. Similar responses were confirmed in T98G cells treated with TMZ, where ATM (Ser 1981) and H2AX (Ser139) together with phospho-Chk2 (Thr68) were strongly expressed at 48 h and were maintained up to 120 h (Supplementary Figures 4A–C). These results suggest that BTZ potentiates the activation of cell cycle checkpoints and accumulation of proteins involved in DNA damage signaling.

Cell cycle kinetics were examined in P3 cells to determine whether cell cycle checkpoints were executed in response to the three treatment regimens. As anticipated, TMZ alone did not substantially alter cell cycle checkpoints nor alter apoptosis threshold in TMZ-resistant P3 cells (Figures 6D,F). BTZ 10 nM treatment, however, led to a significantly reduced cellular portion entering G1 compared to untreated control P3 cells (*p* < .0001), with a concomitant increase in G2/M phase arrest (*p* < .001) as well as increased apoptotic proportions in hypodiploid sub-G1 phase (*p* < .05), Figures 6D,G). *ATG5* ^*−/−*^ P3 cells’ cell cycle progression was not altered by TMZ treatment (Figures 6E,F). However, *ATG5* ^*−/−*^ P3 cells increased cell proportions entering G1 (*p* < .01) and diminished apoptotic fraction in sub-G1 (*p* < .05) compared BTZ treated control cells, essentially reversing the BTZ cytotoxic effect (Figures 6D,E,G). BTZ + TMZ combination treatment also significantly reduced cell fractions in G1 phase compared to untreated P3 control cells (*p* < .0001), and *ATG5* ^*−/−*^ ablation in P3 cells partially re-established the cell proportions entering G1-phase (*p* < .01, Figures 6D,E,H). ATG7shRNA knockdown modestly prevented P3 cell cycle progression to G1 (*p* < .0001) and increased G2/M phase arrest after TMZ (*p* < 0.001, Supplementary Figures S5A,B). Importantly, ATG7shRNA knockdown modestly reversed BTZ induced block in P3 cells’ G1 phase transition (*p* < 0.05, Supplementary Figures S5A,C) while ATG7shRNA knockdown in P3 cells had less effect than BTZ + TMZ treatment (Supplementary Figures S5A, D). Both ATG5−/− and ATG7shRNA knockdown reduced T98G cell fractions entering G1 cell cycle phase after BTZ treatment (*p* < .001 respectively, Supplementary Figures S5F,J,H,L) and increased G2/M arrest compared to untreated cells (*p* < .0001, respectively). Neither ATG5−/− nor ATG7shRNA knockdown in T98G cell had profound effect on responses to BTZ + TMZ combination treatment (Supplementary Figures S5F, J,I,M).

**FIGURE 7:**
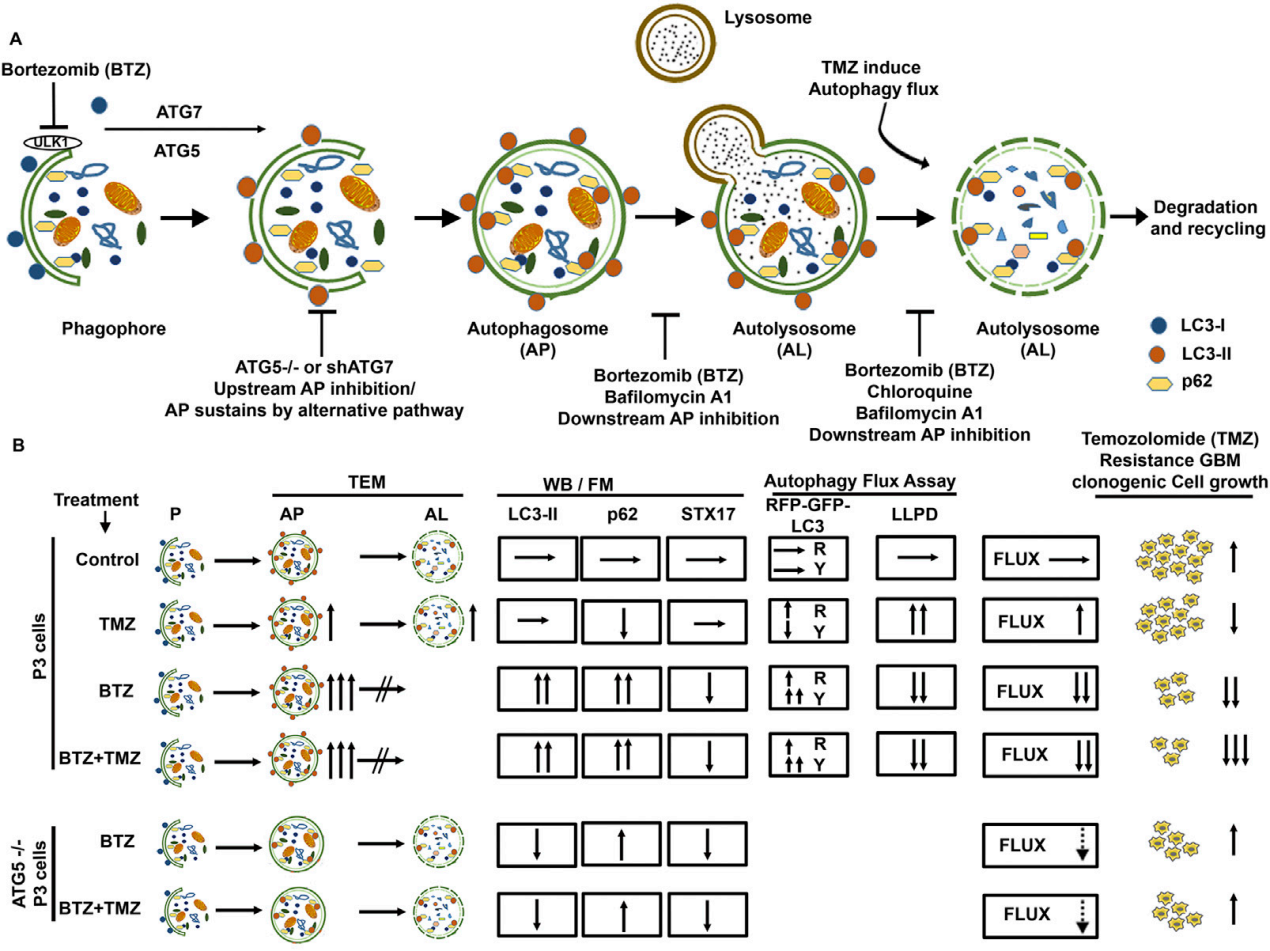
Schematic summary of BTZ mediated inhibition of autophagic flux and its role in abrogating TMZ resistance in GBM cells. **(A)** Autophagosome formation requires the conjugation of cytosolic LC3-I to membrane bound LC3A/B-II and the reaction is catalyzed by autophagy related proteins ATG7 and ATG5. p62 (SQSTM1) is bound to the cargo inside the autophagosome. Subsequent fusion of the autophagosomes (AP) to lysosomes results in autolysosome (AL) formation and degradation of the cargo (autophagic flux). Macromolecules generated from degraded contents of the autophagosome are recycled to the nutrient and energy cellular reservoirs, facilitating cancer cell survival during conditions of stress. To overcome the autophagy-mediated cancer cell survival mechanisms, a number of autophagy inhibitors were tested, including upstream inhibition by knockdown of ATG genes, and downstream inhibition by pharmacological agents like chloroquine, Bafilomycin A1 or proteasome inhibitors. **(B)** TMZ chemotherapy sustains autophagic flux and underlies chemoresistance indicated a high clonogenic survival of GBM cells treated with TMZ. BTZ inhibits autophagic flux by (i) increasing autophagosomes seen on TEM, blocking lysosome-autophagosome fusion (ii) attenuating degradation of long-lived proteins (iii) accumulation of LC3B-II and p62 and reducing STX17 protein lysates, (iv) increased yellow puncta in Autophagy Tandem Sensor RFP-GFP-LC3B assay, and all culminating in reduced clonogenic survival of GBM cells. Pre-treatment of GBM cells with BTZ prior to TMZ treatment effectively inhibits autophagic flux and significantly reduced the clonogenic survival of GBM cells. Knock-out of *ATG5* using CRISPR-Cas9 and Knockdown of ATG7 using shRNA abrogates LC3B-II accumulation and upregulated p62 levels and decreased the number of autophagosomes after BTZ or BTZ + TMZ combination treatment, as more autolysosomes were also observed. This might indicate conventional inhibition of autophagic flux as well as sustained flux by alternative autophagy pathway in *ATG5* ^*−/−*^ and shATG7 GBM cells. While BTZ and BTZ + TMZ combination treatment reduced the clonal survival of TMZ resistant GBM cells, knockdown of *ATG5* or ATG7 rescued GBM cell survival under BTZ or BTZ + TMZ combination treatment indicating that blockade in autophagic flux overcame TMZ resistance and contributed to GBM cell death. AP = Autophagy Flux, P=Phagophore, AP = Autophagosome, AL = Autolysosome, TEM = Transmission electron microscopy, WB = Western Blot, FM = Fluorescence Microscopy, LLPD = Long lived protein degradation assay, R = Red puncta and Y= Yellow Puncta.

This prominent effect of the combination treatment on cell cycle progression was also apparent when examining cell cycle kinetics at the molecular level. Cell fractions expressing phosphorylated histone H3 (Ser10), which is unique to the mitosis phase of cycling somatic cells, were significantly reduced by the combination therapy in P3 cells, compared to TMZ (*p* < .05) or BTZ (*p* < .01) monotherapy (P3 cells, Figures 6I, J; and in T98G, Supplementary Figures S4E). To investigate whether the increased cell death in P3 cells by combination BTZ + TMZ treatment was due to the accumulation of DNA damage in S-phase of the cell cycle, we analyzed phosphorylation of the histone variant H2AX (Ser139) forms pH2AX that marks DNA double-strand breaks. Pre-treatment with BTZ prior to TMZ treatment resulted in significant increase in pH2AX in the S-phase population (*p* = .0127) indicative of accumulation of DNA breaks, that together with the increased phosphorylation of ATM/CHK2 support this hypothesis (Figures 6K,L). Finally, BTZ at lower doses alone or in combination with TMZ induced 4-6-fold increased caspase 3 cleavage in P3 cells (Figures. 6M–O), while caspase 3 was not cleaved following TMZ monotherapy (Figure 6M). Only high dose BTZ 25 nM monotherapy or combination BTZ + TMZ induced cleavage of caspase 3/8 in T98G cells (Supplementary Figures S4G,H). These results indicated that BTZ pre-treatment sensitized GBM cells to TMZ-induced DNA damage and programmed cell death (Figure 7). Perturbing the autophagy machinery with CRISPR-Cas9 *ATG5* ^*−/−*^ and ATG7 shRNA knockdown eliminated the cytotoxic efficacy of BTZ-containing treatments in autophagy-dependent P3 cells (unmethylated MGMT), but not T98G cells (partially methylated MGMT).

Abrogated autophagy flux is postulated to decrease the apoptosis threshold through mechanisms involving the autophagy-regulating transcription factor FOXO3a and p53-upregulated modulator of apoptosis (PUMA). Chemo-resistant cancer cells with high autophagy turnover inhibit transcriptional activation of FOXO3a and PUMA (Thorburn et al., 2014; Fitzwalter et al., 2018; Fitzwalter and Thorburn, 2018). Thus, we investigated the implication of this mechanism in BTZ-mediated blockade of autophagic flux in sensitizing GBM to TMZ. Although no significant differences in PUMA mRNA levels were induced by treatment (Supplementary Figures S5E), FOXO3a mRNA levels were upregulated by approximately 2-fold in BTZ mono- and BTZ + TMZ combination treated P3 GBM cells respectively (*p* < .05) compared to TMZ treatment alone.

## Discussion

In this work, we report that TMZ resistant tumours upregulate autophagic flux, a metabolic process which cancer cells usurp as an adaptive survival mechanism in order to sustain themselves through recycled amino acids and other metabolic precursors from the degraded cytoplasmic components (Simonsen et al., 2008; Galluzzi et al., 2015; Pietrocola et al., 2016), especially during conditions of stress, such as chemotherapy. P3 cells had high basal autophagy flux under steady state and during TMZ treatment, as denoted by low levels LC3A/B-II and p62 (SQSTM1), equal measures of autophagosome and autolysosomes and corroborated by increased degradation of long-lived proteins. TMZ treatment increased the fraction of phospho-histone-H3 positive cells in M-phase cell cycle, and it did not increase apoptotic fractions compared to untreated control cells. These findings are consistent with increased autophagic flux as a pro-survival mechanism. Although both T98G and P3 cells had mutations in *PTEN*, P3 cells harbored additional mutations in *PIK3R2, MAP3K1* and *STAT3* genes that potentially increase downstream PI3K activity and influence autophagy dependence (Jutten et al., 2013). This is important because it has previously been shown that autophagy-dependent tumors respond well to proteasome inhibition (Maycotte et al., 2014).

Further supporting the findings that drug synergy between inhibitors of autophagic flux and anti-neoplastic drugs occur more frequently in autophagy-dependent tumours, we demonstrated that 15 nM BTZ was synergistic with all doses of TMZ, augmenting both P3 and T98G GBM cell killing. T98G cells were, however, less prone to LC3A/B lipidation and cell death by BTZ alone or CQ compared to P3 cells. Perturbation of core autophagy machinery proteins had less robust effects on T98G cells, potentially indicating a less profound dependence on this metabolic process. Specific mutations in critical pathways influence whether a particular cancer type becomes autophagy addicted. Genomic heterogeneity might explain conflicting data regarding efficacy of autophagy inhibitors on different cell types where the same drug combination might conceivably be anti-agonistic in autophagy-independent tumour types (Maycotte et al., 2014; Levy et al., 2017).

Blocking the 26S proteasome with BTZ alone or in combination treatment with TMZ abrogated autophagic flux as evidenced by accumulation of LC3A/B-II that was not further increased in the presence of lysosomal inhibitor BafA1, which prevents autophagosome-lysosome fusion (Yamamoto et al., 1998). Concomitantly increased p62 (SQSTM1) protein in lysates and intracellular compartments from P3 cells, as well as relative numbers of autophagosomes and autolysosomes on electron micrographs confirmed accumulation of autophagosomes. The accumulation of autophagosomes was due to a lack of fusion with lysosomes with subsequent attenuated degradation of cargo. In line with this, STX17 did not colocalize with LC3A/B and the turnover of long-lived proteins attenuated after BTZ + TMZ combination treatment. Through multiple approaches with both static and dynamic consensus methods (Mizushima et al., 2010; Klionsky et al., 2012), including LLPD assays, electron and confocal microscopy to assess the intracellular dynamics of autophagic vesicles, and quantification of the autophagic compartments, we were able to demonstrate consistently that the ratio of autophagosomes to degradative autolysosomes was significantly increased under BTZ + TMZ combination treatment.

BTZ has been contradictorily proposed to induce autophagy (Zhu et al., 2010; Armstrong et al., 2011). Since the induction of LC3A/B-II on immunoblots indicates increased autophagosomes, most studies unilaterally concluded this indicated autophagy induction. Since accumulation of autophagosomes can be due both to increased flux or decreased cargo degradation, one may not conclude on regulation of autophagy flux solely based on LC3A/B lipidation without investigating whether cargo degradation also takes place. Therefore, it is imperative to quantify levels of LC3A/B-II induced by the given treatment and in the presence and absence of the lysosomal protease inhibitor BafA1 (Mizushima and Yoshimori, 2007) and corroborate findings with gold standard dynamic flux assays of long-lived protein degradation (Simonsen et al., 2008). Our results are in line with other studies demonstrating that BTZ blocks the catabolic process of autophagy (Periyasamy-Thandavan et al., 2010; Kao et al., 2014). Treatment of ovarian cancer cells with BTZ, as well as another proteasome inhibitor MG-132, was shown to block autophagic flux by increasing GFP-LC3 puncta, LC3A/B-II and p62 (SQSTM1) protein levels and the number of autophagosomes (Kao et al., 2014). In another study, BTZ treated cells showed higher LC3A/B-II and p62 (SQSTM1) levels and inhibition of the turnover of long-lived proteins in serum starved and rapamycin treated MCF-7 cells, conclusive with blockade of the catabolic process of autophagy (Periyasamy-Thandavan et al., 2010). In agreement with these studies, we determined that BTZ sensitized GBM cells to TMZ by abrogating the autophagic flux at a late stage of the multi-stage process. BTZ treatment strongly induced lipidation of LC3A/B and autophagosome accumulation, similar to treatment with the lysosomal inhibitors BafA1 and CQ. In contrast, depletion of ATG5 or ATG7, proteins that are critical for cargo recruitment and elongation of the phagophore (Komatsu et al., 2001), both abolished lipidated LC3A/B and ATG5^−/−^ rescinded the autophagosome formation in response to BTZ treatment and rescued the cells from BTZ induced death, confirming that BTZ effect was *ATG5* dependent. However, untreated *ATG5* ^*−/−*^ cells did contain more autophagosomes compared to BTZ treated *ATG5* ^*−/−*^ cells, supported by a recent study that demonstrated that cells lacking ATG7 still form autophagosomes through an alternative pathway (Tsuboyama et al., 2016). LC-MS proteomics of the cellular secretome revealed greater levels of MAP1LC3B and p62 (SQSTM1) in supernatants from the untreated *ATG5* ^*−/−*^ cells. Instead of these autophagosomes fusing with lysosomes to degrade cargo, they might have fused directly with the plasma membrane to expel autophagic content to the extracellular space (Cotzomi-Ortega et al., 2018). Hence MAP1LC3B and p62 (SQSTM1) were detected in the secretome but not in the cellular lysate in the ATG5^−/−^ cells.

Most importantly, this failure to increase autophagosome formation under BTZ treatment in *ATG5* and ATG7 knockout P3 and T98G cells rescued them from death by treatment with BTZ alone or in combination with BTZ + TMZ *in vitro.* This *in vitro* finding translates *in vivo* in mice with reduced survival of P3 *ATG5* ^*−/−*^ implanted mice where combination treatment no longer effectively killed the GBM cells, indicating that cell death induced by BTZ is robustly dependent on a functional autophagy machinery. In concurrence, *ATG5* ^*−/−*^ and ATG7 shRNA knockdown reversed the G1 checkpoint arrest, and sub-G1 apoptotic fractions induced by BTZ treatment, particularly in the autophagy-dependent P3 cells. Notwithstanding, the short-term analysis of the cell cycle kinetics was robustly consistent with the findings from long-term colony formation analyses.

In line with this, pharmacological perturbance of the early autophagy components ULK1 and VPS34 with inhibitors MRT68921 and VPS34-IN1, respectively, also abolished lipidated LC3A/B protein, although this had less impact on BTZ-induced cell death. Several explanations can account for the differences in autophagic flux and abolished cell death observed in ATG5 and ATG7 depleted cells *versus* cell treated with inhibitors of the upstream autophagy kinases ULK1/2 and VPS34. The observed block of autophagic flux can be caused by effects of VPS34 on endocytosis. It is interesting to note that ATG5 and ATG7 depletion also reduced cell survival in control cells, although the effects were different from cells treated with ULK1 and VPS34 inhibitors upon co-treatment with BTZ. This might be explained by our finding that ULK1 total and phosphorylated protein levels are diminished in BTZ treated cells, which may cause GBM cell death. A variable ability of some proteasome inhibitors to block macro-autophagy, but not chaperone-induced autophagy, may also play a critical role in the clearance of aggregated proteins or damaged organelles in some cell types (Massey et al., 2008; Koga et al., 2011; Cuervo and Wong, 2014). BTZ is a reversible inhibitor, and we previously showed that the activity of the 20S proteasome returns to baseline within 72 h (Schwartz and Davidson, 2004)^,^ (Rahman et al., 2019). Thus, the period of 120 h encompassing the BTZ + TMZ combination treatment might have allowed partial recovery of autophagic flux.

Another mechanism of TMZ resistance in GBM patients is represented by unmethylated *MGMT* promoter tumours (Xu-Welliver and Pegg, 2002), where the enzyme proficiently repairs the chemotherapy-induced DNA damage. Still, the cells maintain a high apoptosis threshold. We recently showed that BTZ treatment depletes MGMT protein and mRNA and sensitizes GBM cells to TMZ chemotherapy (Rahman et al., 2019). Abrogated autophagic flux would thus represent an additional mechanism by which BTZ kills GBM permitting more rapid DNA damage response by phosphorylation dependent activation of ATM, H2AX, and p53 as well as downstream CHK2 and p21. BTZ + TMZ combination therapy retained elevated phosphorylation of ATM and H2AX induced by BTZ pre-treatment, reduced portion of cells in G1 phase with a concomitant increase in G2/M phase and increased the apoptotic hypodiploid sub G1-fraction. Cell fractions expressing phosphorylated histone H3 (Ser10) were reduced by BTZ + TMZ indicating that BTZ sensitized the cells to TMZ. We have shown in a previous publication that MGMT was downregulated in P3 cells following BTZ treatment (Rahman et al., 2019). We show here that the homologous recombination (HR) pathway is activated (phosphorylation of ATM and CHK2) upon pre-treatment of P3 cells with BTZ prior to TMZ, and double-strand DNA breaks accumulate in the S-phase population of BTZ and TMZ combination treated cells. Taken together these results support a hypothesis where downregulation of MGMT and accumulation of DNA damage in S-phase of combination treated TMZ-resistant glioblastoma cells serve to activate homologous recombination pathway (Ganesa et al., 2022). BTZ abrogated autophagic flux lowered the apoptosis threshold, indicated by increased FOXO3a compared to TMZ treatment, potentially inducing PUMA and apoptosis. These mechanisms enhanced ATM/CHK2 mediated DNA damage and p53-mediated cell cycle arrest (Hirose et al., 2001). Together, our findings are corroborated by a body of work elucidating the molecular crosstalk between autophagy inhibition and apoptosis (Ayna et al., 2012; Warr et al., 2013; Thorburn et al., 2014; Lotsberg et al., 2020).

As we recently demonstrated, BTZ also depleted MGMT protein and mRNA, an additional mechanism that synergized with TMZ to effectively kill GBM cells by enhanced programmed cell death (Rahman et al., 2019). From this study, we further conclude that BTZ pre-treatment induces double-strand break accumulation and homologous recombination (HR) induction upon subsequent TMZ treatment. BTZ treatment has been shown to inhibit autophagy in other cancers, including multiple myeloma, ovarian cancer, endometrial cancer, and hepatocellular carcinoma. Our study brings the novelty that inhibition of autophagy using BTZ synergizes with TMZ treatment to sensitize chemoresistant glioblastoma cells. We have demonstrated mechanistically that BTZ-induced TMZ sensitization is mediated by the abrogation of autophagy in an ATG5-dependent manner. We anticipate uncovering similar mechanisms of efficacy in the patients undergoing treatment in an ongoing phase II clinical trial, NCT03643549 (www.clinicaltrials.gov).

## Data Availability

The datasets presented in this study can be found in online repositories. The names of the repository/repositories and accession number(s) can be found below: https://www.ebi.ac.uk/pride/archive/, PXD021828.

## Glossary

ATCC: American Type Culture Collection
ATG12: Autophagy related 5
ATG16L1: Autophagy related 16 like 1
ATG5: Autophagy related 5
ATG7: Autophagy related 7
ATM: ATM serine/threonine kinase
BafA1: Bafilomycin A1
BTZ: Bortezomib
Cas9: CRISPR associated protein 9
Chk2: Checkpoint kinase 2
CQ: Chloroquine
CRISPR: Clustered regularly interspaced short palindromic repeats
DMEM: Dulbecco’s modified eagle medium
DMSO: Dimethyl sulfoxide
DNA: Deoxyribonucleic acid
ERK: Extracellular signal-regulated kinases
FDA: Food and Drug Administration
FOXO: Forkhead homeobox-type O
GBM: Glioblastoma
GFP: Green fluorescent protein
Gy: Gray
H2AX: H2A histone family member X
IC50: Half maximal inhibitory concentration
IDH1: Isocitrate dehydrogenase 1
LC-MS: Liquid chromatography–mass spectrometry
LLPD: Long-lived protein degradation
MAP1LC3A/B: Microtubule-associated proteins 1A/1B light chain 3B
MAP3K1: Mitogen-activated protein kinase kinase kinase 1
MEK: Mitogen-activated protein kinase kinase
MGMT: O6-methylguanine DNA methyltransferase
μM: Micromolar
nM: Nanomolar
p62 (SQSTM1): Ubiquitin-binding protein p62/Sequestosome-1
PE: Phosphatidylethanolamine
PI: Propidium iodide
PI3K: Phosphoinositide 3-kinases
PIK3C3: Phosphatidylinositol 3-kinase catalytic subunit type 3
PIK3R2: Phosphatidylinositol 3-kinase regulatory subunit beta
PTEN: Phosphatase tensin homolog
PUMA: p53-upregulated modulator of apoptosis
REK: Regional Committees for Medical and Health Research Ethics
RFP: Red fluorescent protein
Ser: Serine
shRNA: Small hairpin ribonucleic acid
SNARE: Soluble NSF attachment receptor
SNV: Single nucleotide variant
STAT3: Signal transducer and activator of transcription 3
STX17: Syntaxin 17
Thr: Threonine
TMZ: Temozolomide
TP53: Tumor protein 53
ULK: Unc-51 like autophagy activating kinase
VPS34: Vacuolar Protein Sorting-associated protein 34
Wt: Wild-type
