# Supplementary figures and images for: Bortezomib abrogates temozolomide-induced autophagic flux through an ATG5 dependent pathway
Source: Front Cell Dev Biol. 2022 Dec 22;10:1022191. doi: 10.3389/fcell.2022.1022191 (PMC9814514; doi:10.3389/fcell.2022.1022191)

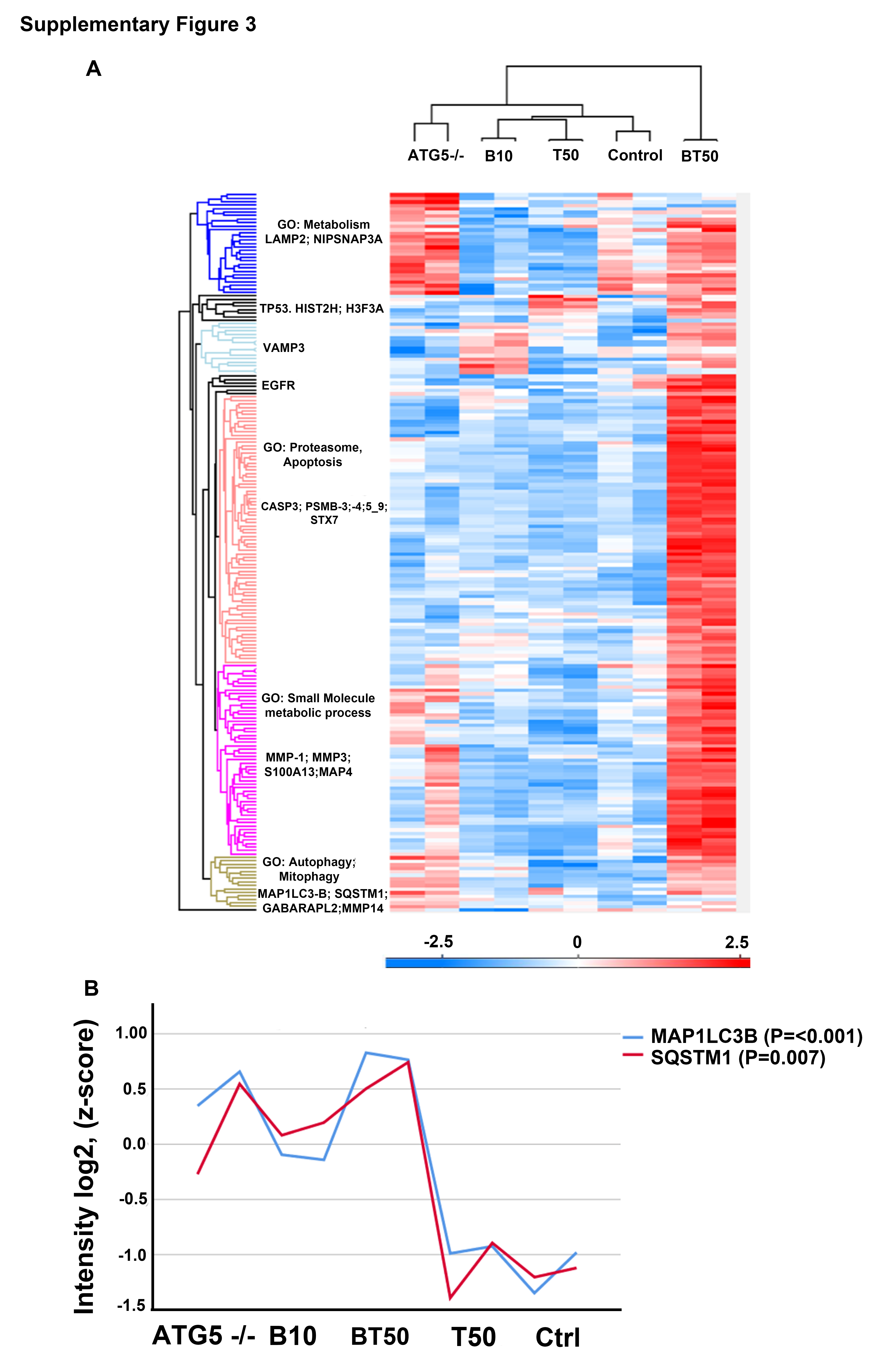

Supplement: Supplementary file 1 [file Image3.TIF]

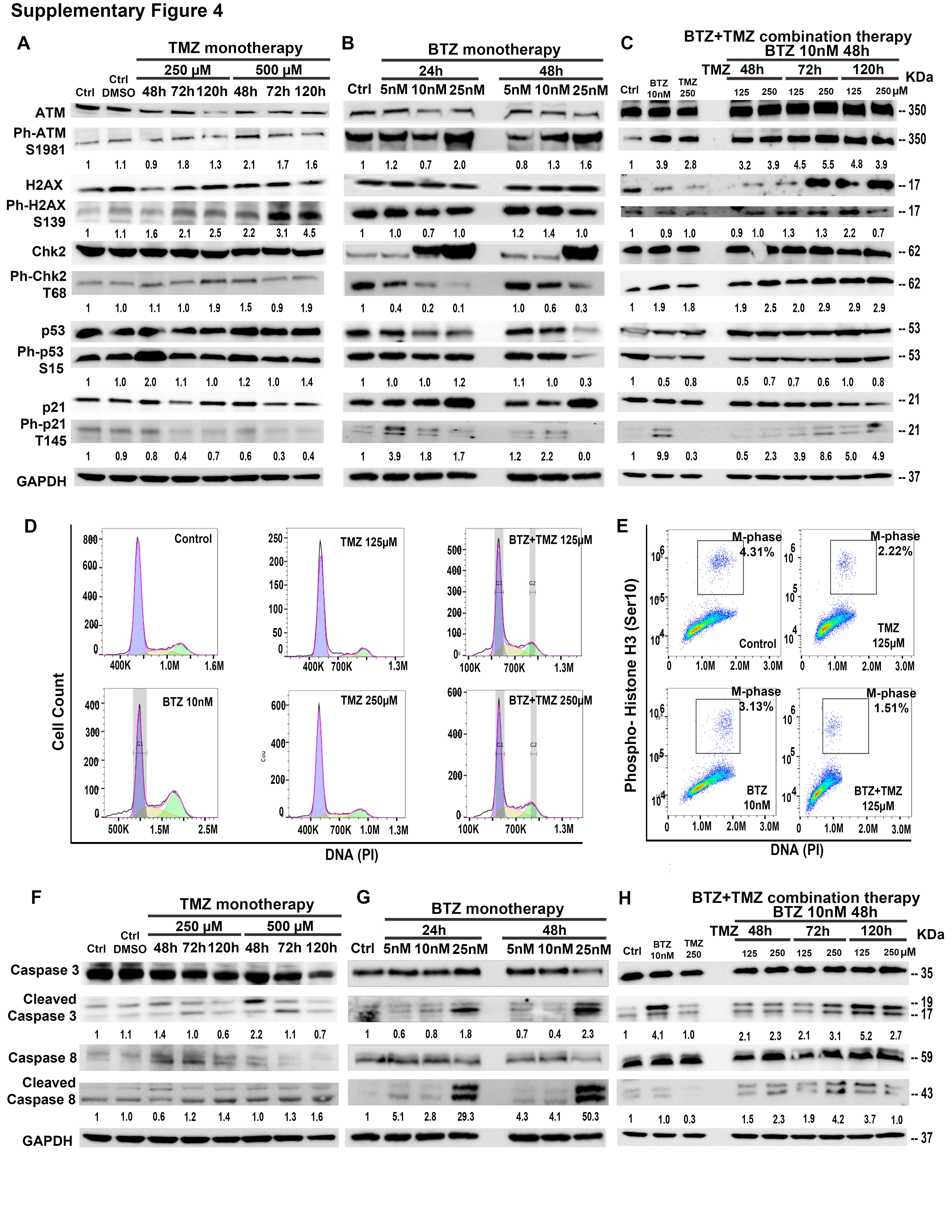

Supplement: Supplementary file 2 [file Image4.jpeg]

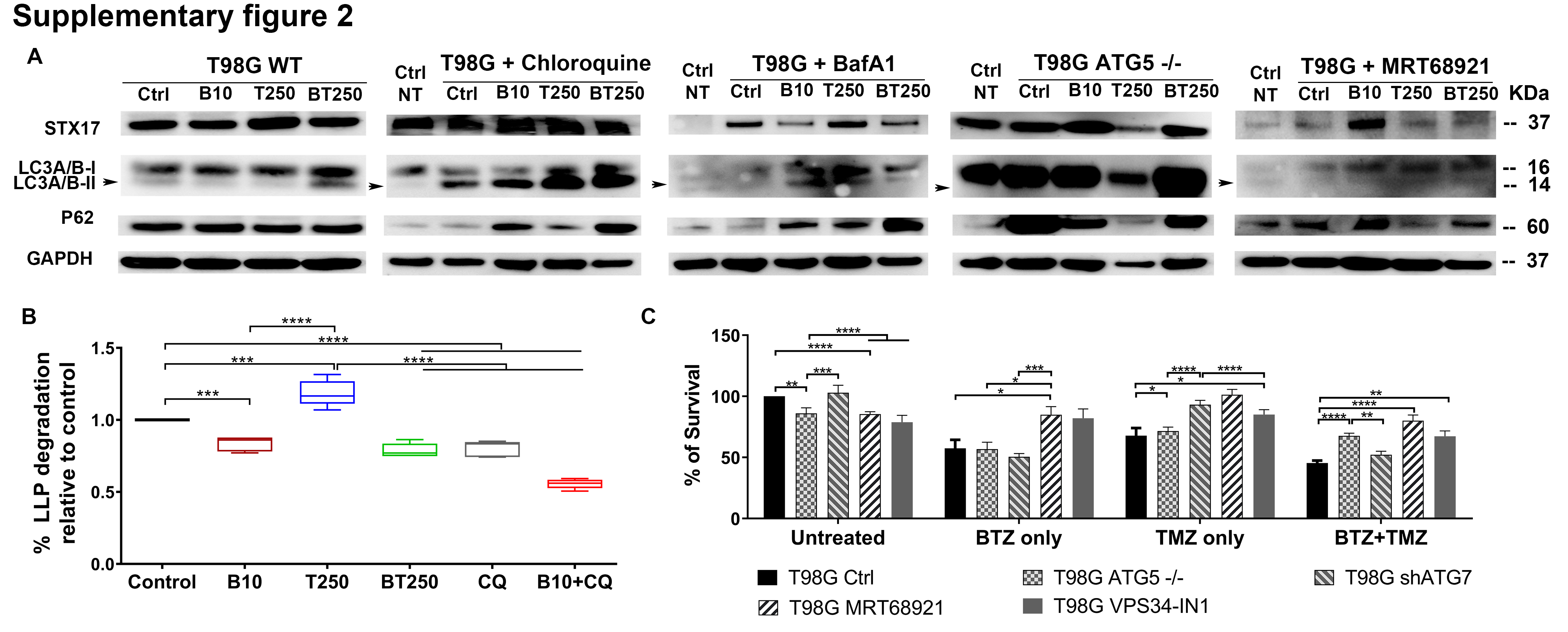

Supplement: Supplementary file 3 [file Image2.TIF]

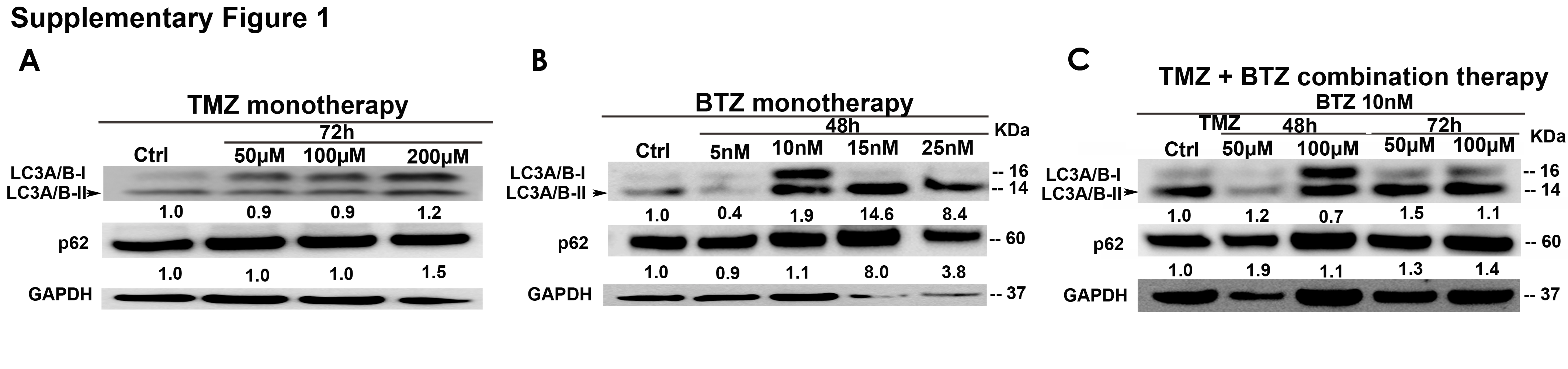

Supplement: Supplementary file 4 [file Image1.TIF]

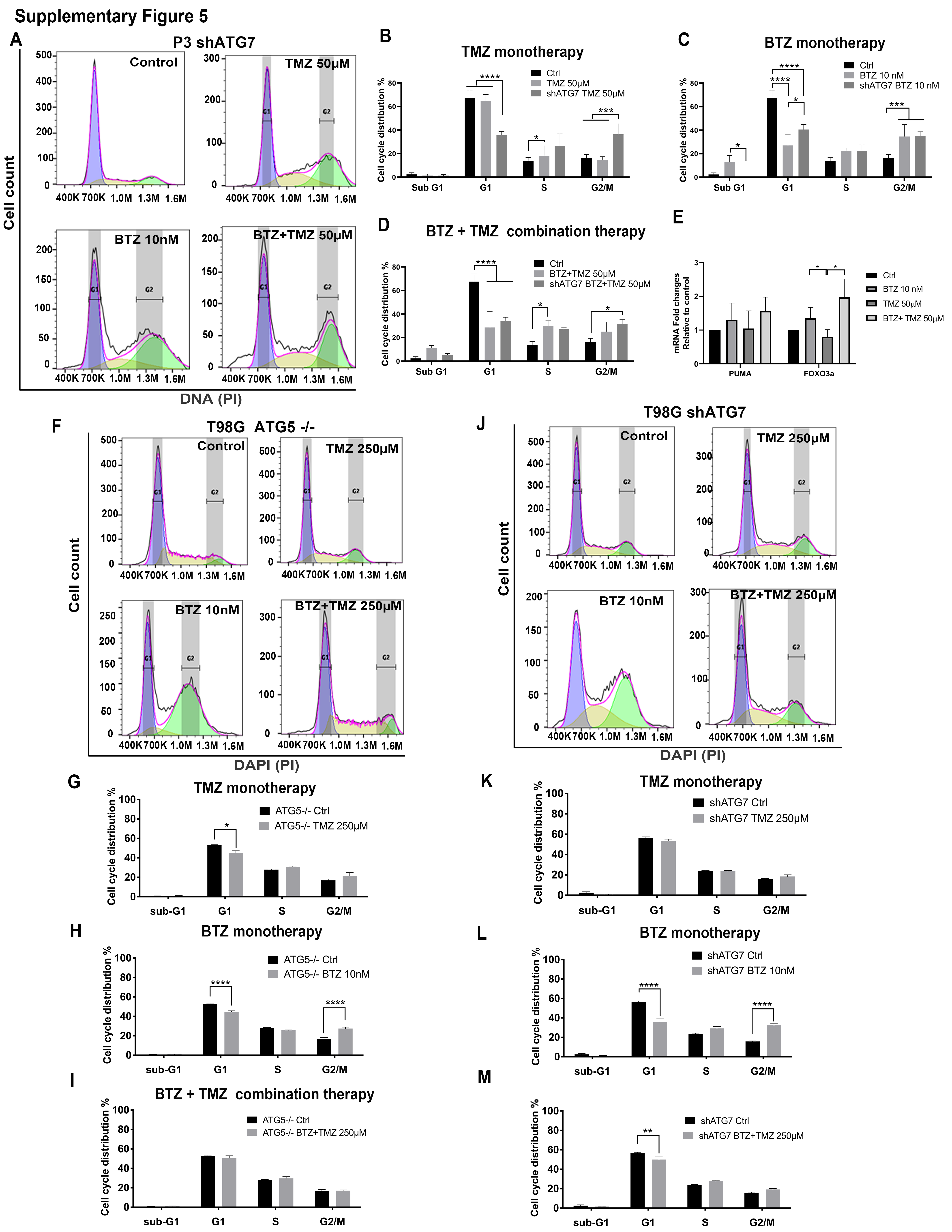

Supplement: Supplementary file 5 [file Image5.TIF]
