# Supplementary material for: Bortezomib abrogates temozolomide-induced autophagic flux through an ATG5 dependent pathway
Source: Front Cell Dev Biol. 2022 Dec 22;10:1022191. doi: 10.3389/fcell.2022.1022191 (PMC9814514; doi:10.3389/fcell.2022.1022191)
Supplement: Supplementary file 6 [file DataSheet1.docx]

**Supplementary results**

*BTZ treatment inhibits TMZ-induced autophagic flux in GBM cells*

Treatment with only BTZ 10nM did not induced detectable levels of LC3-II protein, which was only induced by combination BTZ with 250μM TMZ or addition of chloroquine (CQ) to BTZ treatment (Supplementary Fig. 2A). Neither BafA1, nor ULK1 blockade with MRT68921 could abrogated LC3-II expression, whereas T98G *ATG5^-/-^* cells abrogated LC3-II expression. Modest changes in p62 (SQSTM1) and STX17 were observed, potentially indicating reduced efficacy of BTZ alone to abrogate flux in these cells.

We next investigated whether LC3-II expression after combination BTZ+ TMZ treatment would correlate with degradation of long-lived proteins (LLPD) after treatment as an indication of abrogated autophagic flux. T98G Cells were pulsed with radiolabeled valine and following a chase to deplete short lived proteins and induction of autophagy by starvation, the degradation of long-lived proteins, which is mainly mediated by autophagy, was quantified (Supplementary Fig. 2B). Interestingly, BTZ+TMZ as well as addition of CQ to BTZ treatment or control cells in which LC3-II was expressed, were significantly associated with reduced LLPD compared to control or TMZ treatment (*P* < 0.0001). Although LC3-II was not expressed in BTZ nor TMZ treated cells, LLPD was significantly reduced after BTZ treatment of T98G cells compared to TMZ or no treatment (*P* < 0.001, respectively).

*ATG5^-/-^* ablation, but not ATG7 knockdown, diminished survival of T98 cells. However, treatment with MRT68921 enhanced T98G cell survival (*P* < 0.0001) also during BTZ treatment (*P* < 0.05, Supplementary Fig. 2C). However, in contrast to P3 cells, the VPS34-IN1 inhibitor had no effect on T98G cell survival (Supplementary Fig. 2C). Only blocking PIK3A signaling with VPS34-IN1 inhibitor had marginal effect on clonogenic survival of T98G cells after TMZ treatment (*P* < 0.05, Supplementary Fig. 2C). Combination BTZ+TMZ significantly reduced T98G cell survival. Similarly, to P3 cells, targeting the autophagy machinery with *ATG5^-/-^* ablation, abolished the BTZ effect and rescued the T98G cells from death (*P* < 0.0001, Supplementary Fig. 2C) Taken together, although T98G cells were less dependent on autophagic flux, cell death by abrogated autophagy flux during BTZ combined with TMZ was dependent on ATG5 and upstream mechanisms involving ULK1 and VPS34 mechanisms and less so ATG7.

*BTZ induces DNA damage signaling and cell cycle arrest in TMZ treated T98G GBM cells*

Phosphorylation of p53 (Ser 15) was strongest at 48h following TMZ monotherapy, followed by p21 and phospho-p21 (Thr145) overexpression at 48h indicating cell cycle arrest, (Supplementary Fig. 4A). In contrast to TMZ, an effect of Bortezomib treatment was evident from 24h where in a dose dependent manner, increased pATM (Ser1981), total Chk2 and p21 was apparent. Conversely, the levels of phospho-Chk2 (Thr68), pH2AX, phopsho-p53 and phopsho-p21 at 48h increased at higher BTZ doses indicating disruption of the DNA repair machinery by the proteasome inhibitor (Supplementary Fig. 4B). Combination treatment of TMZ and BTZ increased and sustained upregulation of phospho ATM and phopsho-Chk2, phopsho-H2AX and phopsho-p53 throughout the time course of the treatment (Supplementary Fig. 4C). These results suggest that BTZ potentiates the activation of cell cycle checkpoints and accumulation of proteins involved in DNA damage signaling. Cell cycle kinetics were examined in P3 cells to determine whether cell cycle checkpoints were executed in response to the three treatment regimens.

Similar to P3 cells, TMZ alone did not substantially alter cell cycle checkpoints or apoptosis threshold in TMZ resistant T98G cells (Supplementary Fig. 4D) however, combination BTZ+TMZ treatment led to enhanced portion of cells in G2/M phase arrest and correspondingly 3-fold reduced proportions in M-phase cell cycle (Supplementary Fig. 4E) compared to control. Only combination BTZ+TMZ treatment induced cleavage of 2-fold caspase 3 and 8 in T98G, under condition when LC3-II was expressed, degradation of long-lived proteins was attenuated (Supplementary Fig. 4F-4H). ATG7shRNA knockdown modestly prevented P3 cell cycle progression to G1 (*P* < 0.0001) and increased G2/M phase arrest after TMZ (*P* < 0.001, Supplementary Fig. 5A and 5B). Importantly, ATG7shRNA knockdown modestly reversed BTZ block in P3 cells’ G1 phase transition *(P* < 0.05, Supplementary Fig. 5A and 5C) while ATG7shRNA knockdown in P3 cells had less on effect BTZ+TMZ had (Supplementary Fig. 5A and 5D). Both ATG5^-/-^ and ATG7shRNA knockdown in reduced T98G cell fractions entering G1 cell cycle phase after BTZ treatment (*P* < 0.001 respectively, Supplementary Fig. 5F, 5J, 5H and 5L) and increased G2/M arrest compared to untreated cells (*P* < 0.0001, repsectively). Neither ATG5^-/-^ nor ATG7shRNA knockdown in T98G cell had profound effect on responses to BTZ+TMZ combination treatment (Supplementary Fig. 5F, 5J, 5I and 5M).

**Supplementary methods**

*Preparation of conditional media for LC-MS/MS*

Each tumor line was cultured in T75 flasks to sub-confluence in DMEM complete medium. The media was removed, and cells were washed 5x in PBS and thereafter serum free medium without supplements was added and cells further cultured for 1 hour. After this the cells were additionally washed 5x in PBS, before incubation for 24 hours in fresh serum free media without supplements. The conditioned media (CM) was then collected, centrifuged at 2000×g for 5 min to remove cell debris, followed by sterile filtration (pore size: 0.22 μm, Millipore). The CM was concentrated by ultra-filtration using a centrifugal filter device “Amicon Ultra-15” (Millipore) with centrifugation at 4000 × g for 30 minutes. Protein concentration was measured by using the Qubit Protein Assay.

*Trypsin digestion of conditioned medium*

The CM samples containing 10 μg of protein was buffered with 50mM Tris/1mM CaCl, reduced with 20 mM DTT (7min at 95˚C), alkylated with 50 mM iodoacetamide (1h at RT, (dark), and digested overnight at 37°C with 1:50 enzyme: substrate ratio of sequencing grade trypsin (Promega, Madison, WI). Following digestion, samples were acidified with formic acid and desalted using HLB Oasis SPE cartridges (Waters, Milford, MA). Samples were eluted with 80% acetonitrile in 0.1% formic acid, followed by 100% methanol, and concentrated (speedvac). Peptides were stored at -80°C until use.

*Electrospray ionization liquid chromatography mass spectrometry*

Approximately 0.5μg protein tryptic peptides dissolved in 2% acetonitrile (ACN), 0.5% formic acid (FA), were injected into an Ultimate 3000 RSLC system (Thermo Scientific, Sunnyvale, California, USA) connected online to a Q-Excative HF mass spectrometer (Thermo Scientific, Bremen, Germany) equipped with EASY-spray nano-electrospray ion source source (Thermo Scientific). The sample was loaded and desalted on a pre-column (Acclaim PepMap 100, 2cm x 75µm ID nanoViper column, packed with 3µm C18 beads) at a flow rate of 5µl/min for 5 min with 0.1% TFA. Peptides were separated during a biphasic ACN gradient from two nanoflow UPLC pumps (flow rate of 250 nl/min) on a 25 cm analytical column (PepMap RSLC, 50cm x 75 µm ID EASY-spray column, packed with 2µm C18 beads). Solvent A and B were 0.1% FA (*v/v*) in water and 100% CAN, respectively. The gradient composition was 5% B during trapping (5min) followed by 5-7% B over 0.5min, 7–22% B for the next 61.0min, 24-35% B over 23 min, and 35–90% B over 5min. Elution of very hydrophobic peptides and conditioning of the column were performed during 3 minutes isocratic elution with 90%B and 15 minutes isocratic conditioning with 5% B.

The eluting peptides from the LC-column were ionized in the electrospray and analyzed by the Q-Excative HF. The mass spectrometer was operated in the DDA-mode (data-dependent-acquisition) to automatically switch between full scan MS and MS/MS acquisition. Instrument control was through Q Excative HF Tune 2.8 and Xcalibur 3.1

Survey full scan MS spectra (from m/z 375-1500) were acquired in the Orbitrap with resolution R = 60000 at m/z 200, automatic gain control (AGC) target of 3e6 and a maximum injection time (IT) of 110ms. The 12 most intense eluting peptides above an intensity threshold of 50 000 counts, and charge states 2 to 6, were sequentially isolated to a target value (AGC) of 1e5 and a maximum IT of 110ms in the C-trap, and isolation width maintained at 1.6 m/z (offset of 0.3 m/z), before fragmentation in the HCD (Higher-Energy Collision Dissociation) cell. Fragmentation was performed with a normalized collision energy (NCE) of 28 %, and fragments were detected in the Orbitrap at a resolution of 15 000 at m/z 200, with first mass fixed at m/z 100. One MS/MS spectrum of a precursor mass was allowed before dynamic exclusion for 20s with “exclude isotopes” on. Lock-mass internal calibration (m/z 445.12003) was enabled.

The spray and ion-source parameters were as follows. Ion spray voltage = 1800V, no sheath and auxiliary gas flow, and capillary temperature = 275 °C.

*Data processing and bioinformatic analysis*

MS raw files were analyzed by the MaxQuant software (Version 1.6.1.0), and peak lists were searched against the human SwissProt FASTA database (version May 2019), and a common contaminants database by the Andromeda search engine. As variable modification, methionine oxidation was used and as fixed modification cysteine carbamidomethylation was used. False discovery rate was set to 0.01 for proteins and peptides (minimum length of six amino acids) and was determined by searching a reverse database. Trypsin was set as digestion protease, and a maximum of two missed cleavages were allowed in the database search. Peptide identification was performed with an allowed MS mass deviation tolerance of 20 ppm, and MS/MS fragment ions could deviate by up to 0.5 Da. For accurate intensity-based label-free quantification in MaxQuant [MaxLFQ][1], the type of label was “2″ for LFQ with a minimum ratio count of “2″. Match bewteen runs was used. The mass spectrometry proteomics data have been deposited to the ProteomeXchange Consortium via the PRIDE[2] partner repository with the dataset identifier PXD021828.

*Statistical analysis*

Hierarchical clustering was performed using Perseus software (version 1.6.14.0), using default settings, Euclidian and average linkage. An unsupervised Euclidian hierarchical clustering was performed on the whole dataset. For the multiple samples test an ANOVA with permutation-based FDR (0.05) was performed in Perseus. For a Tukeys post hoc test we applied the SPSS (version 25). For gene ontology String DB (version 11.0) was used with default settings.

**References**

1. Berge, T., et al., *Quantitative proteomic analyses of CD4(+) and CD8(+) T cells reveal differentially expressed proteins in multiple sclerosis patients and healthy controls.* Clin Proteomics, 2019. **16**: p. 19.

2. Perez-Riverol, Y., et al., *The PRIDE database and related tools and resources in 2019: improving support for quantification data.* Nucleic Acids Res, 2019. **47**(D1): p. D442-D450.

**Supplementary Figure Legends**

**Supplementary Figure 1.** Western blot of LC3A/B and p62 (SQSTM1) proteins in lysates from P3 cells after (A) TMZ monotherapy, (B) BTZ monotherapy, (C) BTZ+TMZ combination therapy. GAPDH was used as a loading control and for normalizing densitometry measurements of the blots.

**Supplementary Figure 2.** (A) Western blot of the indicated proteins in T98G lysates after *ATG5* knockout and experimental treatments with and without chloroquine, BafA1 and MRT68921 autophagy inhibitors. GAPDH was used as a loading control. Ctrl NT= non-treated control cells. (B) Percent degradation of long-lived proteins (LLPD) relative to untreated control, quantified as release of ^14^C-valine after indicated treatments for 48 h in the presence or absence of CQ. (C) Clonogenic survival of untreated or treated T98G control, *ATG5* ^-^/^-^, ATG7 knockdown, MRT68921 and VPS34-IN1 treated cells after experimental treatments. Data represents % mean ± S.E.M from at least 10 cells from 2-3 independent experiments; **P* < 0.05, ** *P* < 0.01, *** *P* < 0.001 and **** *P* < 0.0001.

**Supplementary figure 3.** (A) LC-MS/MS proteomics data and Perseus generated heat map of proteins that cluster differentially based on frequency and function in the conditioned media from P3 control cells, P3 cells treated with 10 nM BTZ (B10), 50 µM TMZ (T50) or both (BT50) and from P3 ATG5-/- cells. Proteins that are downregulated are indicated in (blue), upregulated in (red), unchanged (white). (B) Mean ± S.E.M. label free quantification (LFQ) intensity Log2 (z-score) for MAP1LC3B ans SQSTM1 (p62). *P < 0.05, **P < 0.01, ***P < 0.001 and ****P < 0.0001.

**Supplementary figure 4.** Western blot analysis of proteins in DNA damage signaling cascade with total and phospho-proteins in lysates (20 µg) from T98G GBM cells treated with (A) TMZ monotherapy, dose escalation at 48 and 72 h, (B) BTZ monotherapy, dose escalation at 24 and 48 h and (C) combination treatment with BTZ 10 nM and TMZ, dose escalation at 48, 72 and 120 h. Data represents at least two independent experiments. GAPDH was used as a loading control and for normalizing densitometry measurements of the blots. (D) DNA histograms showing cell cycle distribution of propidium iodide (PI) stained T98G cells, control and after respective treatment. (E) Percent T98G cells positive for phospho-histone H3 in M-phase of the cell cycle after respective treatment. Western blot analysis of total and cleaved caspase 3/8 in protein lysates from T98G GBM cells treated with (F) TMZ monotherapy, dose escalation at 48 and 72 h, (G) BTZ monotherapy, dose escalation at 24 and 48 h and (H) combination treatment with BTZ 10 nM and TMZ dose escalation at 48, 72 and 120 h. GAPDH was used as a loading control and for normalizing densitometry measurements of the blots. GAPDH blots in F-H is from the same samples/experiments used in A-C.

**Supplementary Figure 5.** DNA histograms showing cell cycle distribution of propidium iodide (PI) stained P3 (A) shATG7 cells, untreated control and after respective treatment. Quantification of percent P3 (B-D) shATG7 cells in respective cell cycle phases as indicated treatment for TMZ monotherapy, BTZ monotherapy and BTZ+TMZ therapy. (E) FOXO3a mRNA fold change after indicated treatments in P3 cells. DNA histograms showing cell cycle distribution of propidium iodide (PI) stained T98G (F), ATG5 -/- and (J) ATG7shRNA cells, untreated control and after respective treatment. Quantification (%) of T98G (G-I) ATG5 -/- and (K-M) ATG7 shRNA cells in respective cell cycle phases as indicated treatment for TMZ monotherapy, BTZ monotherapy and BTZ+TMZ therapy. Data represents % mean ± S.E.M from 2-3 independent experiments; **P* < 0.05, ** *P* < 0.01, *** *P* < 0.001 and **** *P* < 0.0001.

**Supplementary Table 1: List of antibodies**

All dilutions are for western blotting except where indicated for IF (Immunofluorescence)

| Antibody | Cat. number (Manufacturer) | RRID | Dilution |
| --- | --- | --- | --- |
| Rabbit Anti-ATM | 2873S (Cell Signaling Technology, Massachusetts, USA) | AB_2062659 | 1:1000 |
| Mouse Anti-phospho-ATM S1981 | 200-303-400 (Rockland Immunochemicals Limerick, USA) | AB_217870 | 1:1000 |
| Rabbit Anti-ATM (phospho S1981) [EP1890Y] | ab81292 (Abcam, UK) | AB_1640207 | 1:1000 |
| Mouse Anti-H2AX | 7631S (Cell Signaling Technology) | AB_10860771 | 1:1000 |
| Rabbit Anti-phospho-H2AX Ser139 | 07-164 (Merck Millipore, Burlington, Massachusetts, USA) | AB_310406 | 1:250 |
| Mouse Anti-phospho-Histone H2A.X (Ser139), clone JBW301 | 05-636 (Merck Millipore, Burlington, Massachusetts, USA) | AB_309864 | 1:1000 |
| Rabbit Phospho-histone H3 (Ser10) (D2C | 3465S (Cell Signaling Technology) | AB_10695860 | 1:500 |
| Rabbit Anti-p53 | SC-126 (Santa Cruz Biotechnology Dallas, TX, USA) | AB_628082 | 1:1000 |
| Rabbit Anti-phospho-p53 S15 | 9284S (Cell Signaling Technology) | AB_331464 | 1:1000 |
| Rabbit Anti-Chk2 | 2662 (Cell Signaling Technology) | AB_2080793 | 1:1000 |
| Rabbit Anti-phospho-Chk2 Thr68 | 2661L (Cell Signaling Technology) | AB_331479 | 1:1000 |
| Rabbit Anti-p21 | 2947S (Cell Signaling Technology) | AB_10829764 | 1:1000 |
| Rabbit Anti-phospho-p21 T145 | SC-20220-R (Santa Cruz biotechnology) | AB_2077693 | 1:250 |
| Rabbit Anti-Caspase 3 | 9662S (Cell Signaling Technology) | AB_331439 | 1:1000 |
| Rabbit Anti-Cleaved caspase 3 | 9664S (Cell Signaling Technology) | AB_2070042 | 1:500 |
| Rabbit Anti-Caspase 8 | 4790S (Cell Signaling Technology) | AB_10545768 | 1:1000 |
| Rabbit Anti-Cleaved caspase 8 | 9496S (Cell Signaling Technology) | AB_561381 | 1:500 |
| Rabbit Anti-p62 | 5114S (Cell Signaling Technology) | AB_10624872 | 1:1000 |
| Rabbit Anti-LC3 | 12741S (Cell Signaling Technology) | AB_2617131 | 1:1000 |
| Mouse Anti-LC3 | ab243506 (Abcam, UK) | - | 1:100 (IF) |
| Rabbit Anti-ATG5 | 12994S (Cell Signaling Technology) | AB_2630393 | 1:1000 |
| Rabbit Anti-ULK1 | 8054S (Cell Signaling Technology) | AB_11178668 | 1:1000 |
| Rabbit Anti-Phospho-ULK1 Ser757 | 14202S (Cell Signaling Technology) | AB_2665508 | 1:1000 |
| Rabbit Anti-ATG7 | ab52472 (Abcam) | AB_867756 | 1:1000 |
| Rabbit Anti STX17 | PA5-40127 (Thermo Fisher Scientific) | AB_2608436 | 1:1000  1:100 (IF) |
| Rabbit Anti-GAPDH | 5174S (Cell Signaling Technology) | AB_10622025 | 1:1000 |
| Rabbit Anti-p62/SQSTM1 | Ab566416 (Abcam) | - | 1:100 (IF) |
| Goat Anti Rabbit IgG- HRP | 31460 (Thermo Fisher Scientific) | AB_228341 | 1:10000 |
| Goat Anti Mouse IgG- HRP | sc-2031 (Santa Cruz Siotechnology) | AB_631737 | 1:10000 |
| Goat anti-Mouse IgG (H+L), Alexa Fluor Plus 488 | A32723(Thermo Fisher Scientific) | AB_2633275 | 1:200 (IF) |
| Goat anti-Mouse IgG (H+L), Alexa Fluor Plus 488 | A32732 (Thermo Fisher Scientific) | AB_2633281 | 1:200 (IF) |
